# Tomato LysM Receptor-Like Kinase SlLYK12 Is Involved in Arbuscular Mycorrhizal Symbiosis

**DOI:** 10.3389/fpls.2018.01004

**Published:** 2018-07-11

**Authors:** Dehua Liao, Xun Sun, Ning Wang, Fengming Song, Yan Liang

**Affiliations:** College of Agriculture and Biotechnology, Zhejiang University, Hangzhou, China

**Keywords:** innate immunity, chitin, chitin elicitor receptor kinase (CERK1), arbuscular mycorrhiza (AM) symbiosis, LysM receptor-like kinase (LYK), *Solanum lycopersicum*, SlLYK1, SlLYK12

## Abstract

Arbuscular mycorrhiza (AM) is a widespread symbiotic relationship between plants and fungi (Glomeromycota), which improves the supply of water and nutrients to host plants. AM symbiosis is set in motion by fungal chitooligosaccharides and lipochitooligosaccharides, which are perceived by plant-specific LysM-type receptor kinases (LYK). In rice this involves OsCERK1, a LYK also essential for chitin triggered innate immunity. In contrast in legumes, the CERK1 homologous gene experienced duplication events resulting in subfunctionalization. However, it remains unknown whether this subfunctionalization is legume-specific, or has occurred also in other dicot plant species. We identified four CERK1 homologs in tomato (SlLYK1, SlLYK11, SlLYK12, and SlLYK13) and investigated their roles in chitin signaling and AM symbiosis. We found that knockdown of SlLYK12 in tomato significantly reduced AM colonization, whereas chitin-induced responses were unaffected. In contrast, knockdown of SlLYK1 resulted in reduced responses to chitin, but did not alter responses to AM fungi. Moreover, ectopic overexpression of SlLYK1 and SlLYK13 in *Nicotiana benthamiana* induced cell death, whereas SlLYK12 overexpression did not. Based on our results and comparison with rice OsCERK1, we hypothesize that OsCERK1 orthologs in tomato underwent gene duplication, leading to the subfunctionalization of immunity and symbiosis.

## Introduction

*N*-acetyl-D-glucosamine (GlcNAc)-containing molecules are important microbial signaling factors, and include chitin from pathogenic fungi, peptidoglycan from pathogenic bacteria, Nod factors from symbiotic rhizobia, and Myc factors from symbiotic arbuscular mycorrhizal (AM) fungi. These molecules mediate the initiation of either plant innate immune functions or symbiotic pathways (Liang et al., 2014; Zipfel and Oldroyd, 2017).

Chitin, a polymer of GlcNAc, is the major component of the fungal cell wall. When fungi infect plants, plants secrete chitinases to hydrolyze chitin, producing chitooligosaccharides among which those with degrees of polymerization (dp) between 6 and 8 elicit plant immune responses. Such immune responses include the elevation of cytosolic calcium, production of reactive oxygen species (ROS), induction of defense-related gene expression, callose deposition, and pathogen growth restriction (Boller and Felix, 2009; Dodds and Rathjen, 2010). In *Arabidopsis thaliana*, chitin is primarily recognized by LysM RECEPTOR KINASE5 (AtLYK5), which has an inactive kinase domain (Cao et al., 2014). After perception of chitin, AtLYK5 forms a heterotetramer complex with CHITIN ELICITOR RECEPTOR KINASE1 (AtCERK1), activating the AtCERK1 intracellular kinase domain and downstream immune responses (Cao et al., 2014; Zipfel and Oldroyd, 2017; Desaki et al., 2018). In rice, CHITIN ELICITOR-BINDING PROTEIN (OsCEBiP), which has no kinase domain, play the major role in chitin perception, and transduces signals via a similar mechanism of complex formation with OsCERK1(Shimizu et al., 2010; Hayafune et al., 2014). Recently, a *Lotus japonicus* ortholog of CERK1, LjCERK6, required for chitin responses was identified (Bozsoki et al., 2017). Thus, CERK1 kinase activity is a key factor in the induction of chitin-induced immune responses.

In contrast to chitin oligomers, acylated chitooligosaccharides (so called lipochitooligosaccharides, LCOs) can act as signaling molecules triggering symbiosis; for example, Nod factors from rhizobia. Nod-LCOs are also recognized by the LysM-containing receptors, called NFR1-NFR5 (NF RECEPTOR1 and 5) complex in *L. japonicus*, and LYK3-NFP (NF PERCEPTION) complex in *Medicago truncatula* (Ben Amor et al., 2003; Limpens et al., 2003; Madsen et al., 2003; Radutoiu et al., 2003; Broghammer et al., 2012). Similar to the chitin recognition model in *Arabidopsis*, the kinase domain of NFR5/NFP is inactive, and the Nod-LCO signal is transduced via NFR1/LYK3 kinase activation (Kelly et al., 2017; Zipfel and Oldroyd, 2017; Desaki et al., 2018).

GlcNAc-containing molecules are also important signals (Myc factors) for AM symbiosis, a widespread symbiotic relationship occurring between fungi in the phylum Glomeromycota and 80% of terrestrial plant species under phosphorus- or nitrogen-limiting conditions (Schussler et al., 2001; Nouri et al., 2014). The AM symbiosis is characterized by the formation of arbuscule structures in the root cortex, the main site for nutrient exchange between the two symbiotic partners (Harrison, 2012). Myc factors are thought to be a mixture of short-chain chitin (dp = 3–5) and Myc-LCOs (Maillet et al., 2011; Genre et al., 2013). Perception of Myc factors activates the common symbiosis signaling pathway shared with Nod factor signaling in potential host plants, leading to the colonization of AM fungi in root epidermal cells (Oldroyd, 2013). Given the molecular similarities between Myc-LCO and Nod-LCO, as well as the shared common symbiosis signaling pathway, it has been proposed that Nod factor recognition may have evolved from Myc factor receptors (De Mita et al., 2014). Indeed, the ortholog of NFP/NFR5 in *Parasponia andersonii*, the only non-leguminous plant that can establish symbiosis with rhizobia, is required for both AM and rhizobial symbioses (Op den Camp et al., 2011). Similarly, knockdown of the ortholog of NFP/NFR5 in tomato (SlLYK10) affects AM colonization (Buendia et al., 2016), whereas the ortholog of NFP/NFR5 in rice is not required for AM symbiosis (Miyata et al., 2014). Interestingly, OsCERK1, the ortholog of NFR1/LYK3 in rice, is involved in both chitin and Myc factor signaling (Zhang et al., 2015; Miyata et al., 2016; Carotenuto et al., 2017). However, this dual function of CERK1 in AM symbiosis and chitin-triggered innate immunity is separated of two paralogous genes in legumes (Bozsoki et al., 2017). This raises the question whether such subfunctionalization upon gene duplication is specific for the legume family, or may have occurred also in non-related dicot species; e.g., tomato.

In this study, we identified the orthologs of the CERK1 subclade in tomato, and investigated their function by knocking down their expression. We found that knockdown of *SlLYK12* significantly reduced AM colonization; however, the chitin responses of these plants were similar to those of controls. In contrast, knockdown of *SlLYK1* resulted in reduced responses to chitin, but normal responses to AM fungi. In addition, we found that ectopic overexpression of *SlLYK1* and *SlLYK13* in *Nicotiana benthamiana* caused cell death; however, *SlLYK12* overexpression did not. Taken together, these results suggest a hypothesis whereby an ancestor of *CERK1* with dual function in both immunity and symbiosis gave rise to multiple molecules during evolution through gene duplication in tomato, among which SlLYK1 and SlLYK12 were sub-functionalized for a role in immunity and symbiosis, respectively.

## Materials and Methods

### Plant Materials and Growth Conditions

*Solanum lycopersicum* L. cv Zheza 809 was used for all experiments. For virus-induced gene silencing (VIGS) experiments, seedlings were grown in a plant growth room at 22°C with a 16 h photoperiod. For *Rhizophagus irregularis* inoculation, plants were grown at 25°C with a 16 h photoperiod.

### RNA Isolation and Quantitative RT-PCR (qRT-PCR)

Total RNA was isolated using TRIzol reagent (Invitrogen, Waltham, MA, United States). RNA samples were treated with DNase I to eliminate potential contamination with genomic DNA. qRT-PCR was performed on an Applied Biosystems Plus Real-Time PCR System (ABI, Foster city, CA, United States) using a SYBR premix Ex Taq kit (Takara, Mountain View, CA, United States). Primers are listed in Supplementary Table 1.

### Gene Cloning and Plasmid Construction

The primers used for gene cloning are listed in Supplementary Table 1. Full length coding sequences for ectopic overexpression and fragments for VIGS experiments were amplified from cDNA. The amplified sequences were cloned into the pDONR/Zeo plasmid by BP cloning (Invitrogen, Waltham, MA, United States). After verification by sequencing, resultant plasmids were used for LR cloning into the destination plasmids pTRV2 for VIGS and pMDC83 for overexpression in *N. benthamiana*. All plasmids were introduced into *Agrobacterium tumefaciens* strain GV3101 by electroporation.

### Gene Silencing Assay Using *Tobacco rattle virus* (TRV)

Agrobacteria carrying pTRV2-*GUS* (*β-GLUCURONIDASE*, a negative control), pTRV2-*NbPDS* (*PHYTOENE DESATURASE*, a positive control for monitoring the progress of gene silencing), pTRV2-*SlLYK1, 12, 13*, and pTRV1 were cultivated in YEP medium (10 g/L yeast extract, 10 g/L peptone, 5 g/L NaCl, 50 μg/mL kanamycin, 50 μg/mL rifampicin, and 25 μg/mL gentamicin) for 36 h at 28°C. Cultures were passaged in fresh medium at a dilution of 1:100 and cultivated for a further 8 h. After adjusting the concentration to OD_600_ = 1.5, each pTRV2 construct was mixed with pTRV1 (1:1) in infiltration buffer (10 mM MgCl_2_, 10 mM MES, 150 μM acetosyringone, pH 5.7). The agrobacterial mix was infiltrated into the abaxial surface of 10-day-old tomato seedlings. Gene silencing efficiency and specificity were determined 4 weeks after agrobacterial infiltration. At least six individual seedlings were analyzed for each construct.

### Mycorrhizal Inoculation

*Rhizophagus irregularis* was purchased from the Institute of Plant Nutrition and Resources, Beijing Academy of Agriculture and Forestry Sciences, and propagated using *N. tabacum* as the host in 4 L pots containing sand. Plants were watered with a solution without added phosphorous every week. Four months later, the sand containing *R. irregularis* was harvested and dried to obtain mycorrhizal inoculums. For mycorrhizal inoculation, after VIGS infiltration, each tomato plant was transferred to a 4 L pot containing sand including 4 g of sand-based mycorrhizal inoculums at the base of the roots.

### Detection of Mycorrhizal Colonization

Roots were cleared with 10% (w/v) KOH for 1 h at 90°C, acidified with 2% HCl for 5 min, and then stained with trypan blue (0.5 mM). Mycorrhizal colonization was observed under a light microscope (Nikon, Tokyo, Japan). The rate of root colonization was quantified using the grid line intersect method at 3 and 6 weeks after mycorrhizal inoculation, and calculated as the ratio of intersects with hyphopodia, intracellular hyphae, arbuscules and vesicles over all root intersects (100 intersects per plant) × 100 (Vierheilig et al., 1998).

### Reactive Oxygen Species (ROS) Assay

Leaf disks (diameter, 0.5 cm) were punched and incubated in water for at least 8 h. After addition of water containing 1.25 μM L-012 chemiluminescent probe (Wako Chemicals USA, Richmond, VA, United States), 20 μg/mL horseradish peroxidase, and 500 nM chitooctaose (IsoSep, Tullinge, Sweden), chemiluminescent signals were immediately recorded using a Photek camera (HRPCS5; Photek Ltd., East Sussex, United Kingdom) for 30 min.

### Detection of Cell Death in *N. benthamiana* Leaves

Cell death in *N. benthamiana* leaves was detected by trypan blue staining. Excised leaves were vacuum-infiltrated with trypan blue solution (2.6 mM) for 30 min, and incubated for a further 8 h. Leaves were then destained in a solution containing ethanol and glycerol at a ratio of 9:1 at 65°C for 30 min.

### Antibodies and Immunoblot Analysis

Immunoblot analysis was performed as previously described (Liao et al., 2017) using anti-phospho-p44/p42 MAP kinase antibody (Cell Signaling Technology, Danvers, MA, United States).

### Bioinformatics Analysis

The amino acid sequences of LYK genes are listed in Supplementary Table 2. Multiple sequence alignments were performed using the ClustalX program (version 1.83) with default gap penalties. An approximately maximum-likelihood tree was constructed using the FastTree program with default parameters^1^.

## Results

### SlLYK12 Is Required for AM Symbiosis

To identify orthologs of OsCERK1/AtCERK1 in tomato, we generated a phylogenetic tree using the full length amino acid sequences of *S. lycopersicum* LYKs. The results showed that four genes from *S. lycopersicum, SlLYK1, SlLYK11, SlLYK12*, and *SlLYK13*, were clustered into one clade with *AtCERK1*, similar to the tree constructed by Buendia et al. (2016) using intracellular region sequences. Previously generated RNA sequencing data indicate that the *SlLYK1* gene is expressed at similar levels in roots and leaves, and the *SlLYK12* and *SlLYK13* genes were each primarily expressed in roots and leaves, respectively (Supplementary Figure 1A) (Tomato Genome, 2012). We confirmed these results using qRT-PCR (Supplementary Figure 1B). The expression level of *SlLYK11* was much lower in both roots and leaves compared to the other three genes (Supplementary Figure 1). In addition, we were unable to amplify the predicted full-length coding sequence of the *SlLYK11* gene, even using cDNA extracted from chitooctaose (CO8)-treated leaves and AM-inoculated roots; therefore, we did not perform further analysis of the *SlLYK11* gene.

**FIGURE 1:**
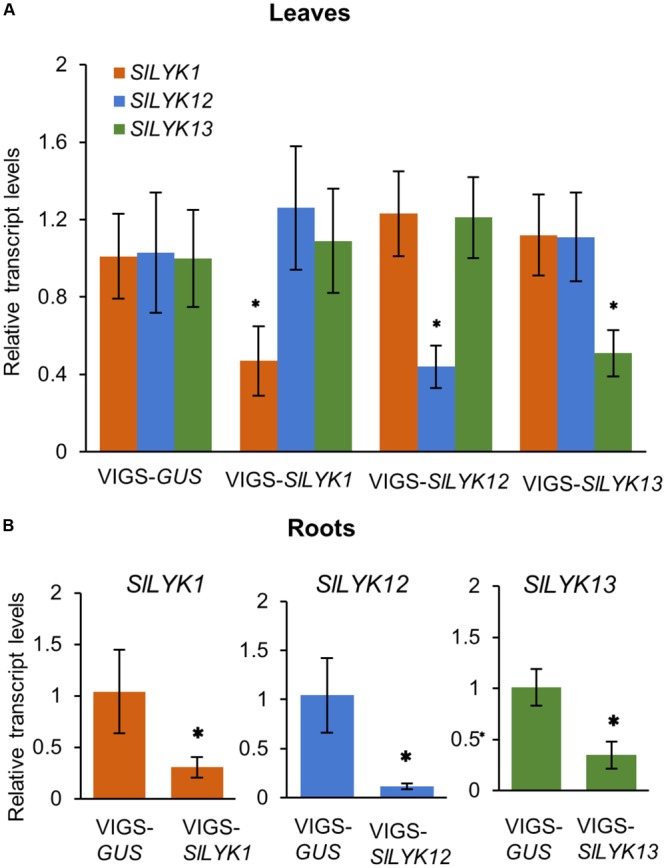
Each endogenous gene of CERK1 family in tomato was specifically silenced by its respective VIGS construct. **(A)** The relative transcript levels of *SlLYK1, SlLYK12*, and *SlLYK13* in leaves infiltrated with VIGS-*SlLYK1, -12, -13*, or -*GUS* (control). **(B)** Relative transcript levels in roots. RNA was extracted from leaves and roots 4 weeks after leaf infiltration with *Agrobacterium tumefaciens* carrying the indicated constructs. Transcript levels were detected using qRT-PCR. Data are expressed as means ± SD from three biological replicates. Asterisks indicate significant differences from the VIGS-*GUS* control (Student’s *t-*test: ^∗^*P* ≤ 0.05).

**FIGURE 2:**
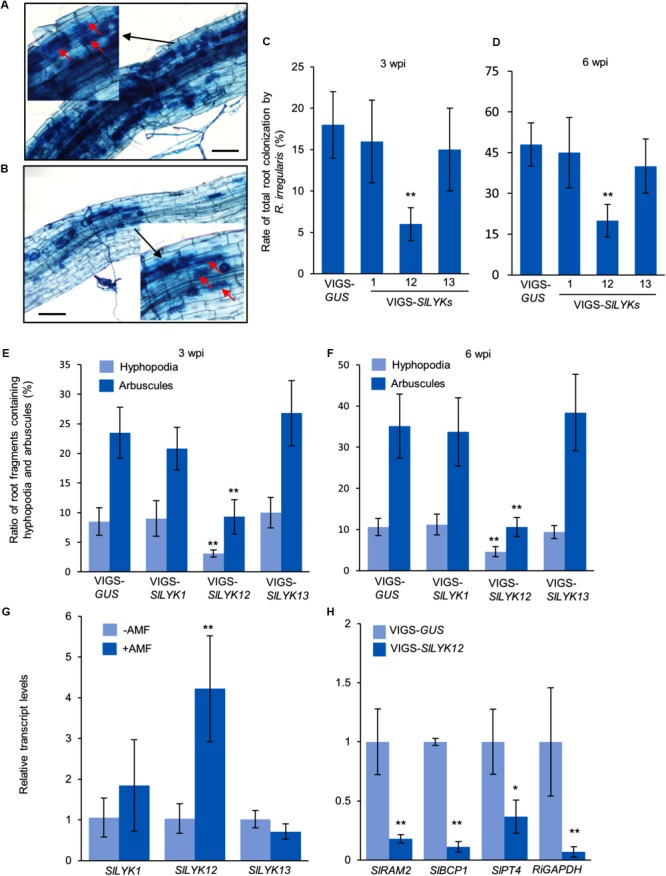
SlLYK12 is required for AM symbiosis. **(A,B)** Images of roots inoculated with *Rhizophagus irregularis* after infiltration with VIGS-*GUS* control **(A)** and VIGS-*SlLYK12* constructs **(B)**. **(C,D)** Rate of total root colonization by *R. irregularis* 3 and 6 weeks post inoculation (wpi), respectively. **(E,F)** Ratio of root fragments containing hyphopodia and arbuscules at 3 and 6 wpi, respectively. Plants were inoculated with *R. irregularis* 4 weeks after infiltration with VIGS-*SlLYK1, -12, -13*, or VIGS-*GUS* constructs. Roots were stained with trypan blue to visualize fungal structures. The rate of root colonization was calculated using the grid line intersect method (100 intersects for each plant and six plants for each construct). Images were taken 6 wpi. Arbuscules are indicated by arrows. **(G)** The expression levels of *SlLYK1, SlLYK12*, and *SlLYK13* in roots inoculated with or without *R. irregularis* (AMF). **(H)** The expression levels of mycorrhizal responsive genes and fungal housekeeping gene. RNA was extracted from the roots of VIGS-*SlLYK12*-infiltrated plants and controls. Gene expression was detected by qRT-PCR. Data are expressed as means ± SD from three biological replicates. Asterisks indicate significant differences from the VIGS-*GUS* control (Student’s *t-*test: ^∗^*P* ≤ 0.05, ^∗∗^*P* ≤ 0.01).

To study the function of *SlLYK1, SlLYK12*, and *SlLYK13*, we silenced these genes individually using a VIGS approach, which is a powerful tool for the study of AM symbiosis in the tomato (Buendia et al., 2016). As the sequence similarity between these three genes is 74%, we designed three sets of primers for each gene and each amplified region was fused to the VIGS vector. The transcript levels of *SlLYK1, SlLYK12*, and *SlLYK13* were detected by qRT-PCR in VIGS leaves 4 weeks after agrobacterial infiltration. The best sets of primers were chosen according to the specificity and efficiency of gene silencing; for example, *SlLYK1* gene expression was down-regulated 50% in VIGS- *SlLYK1* leaves compared to VIGS-*GUS* (*β-GLUCURONIDASE*) control, but the transcript levels of *SlLYK12* and *SlLYK13* did not show significant differences (**Figure 1A**). After confirming the silencing effectiveness in roots (**Figure 1B**), roots were inoculated with *R. irregularis* and grown for another 3 and 6 more weeks. To observe mycorrhizal colonization, roots were harvested and stained with trypan blue. Arbuscules could be observed in plants infiltrated with all constructs (**Figures 2A,B**). The rate of total root colonization including hyphopodia, intracellular hyphae, arbuscules, and vesicles was calculated using the grid line intersect method (Vierheilig et al., 1998). We found that VIGS-*SlLYK12*- infiltrated plants exhibited a more than 50% reduction of mycorrhizal colonization, whereas those infiltrated with VIGS-*SlLYK1* and -*SlLYK13* did not exhibit significant differences from control plants (**Figures 2C,D**). Similarly, the percentage of hyphopodia and arbuscules were significantly reduced in VIGS-*SlLYK12*- infiltrated plants (**Figures 2E,F**). Consistent with these results, levels of the *SlLYK12* transcript were increased fourfold after *R. irregularis* inoculation, whereas those of *SlLYK1* and *SlLYK13* were not (**Figure 2G**).

To confirm that *SlLYK12* is required for the development of AM symbiosis, we determined the expression levels of a fungal housekeeping gene *GLYCERALDEHYDE 3-PHOSPHATE DEHYDROGENASE* (*RiGAPDH*), and three AM responsive genes in tomato, including *REDUCED ARBUSCULAR MYCORRHIZATION2* (*SlRAM2*, an early signaling gene in the common symbiosis pathway), *BLUE COPPER-BINDING PROTEIN1* (*SlBCP1*, a gene induced in arbuscule-containing regions), and *PHOSPHATE TRANSPORTER4* (*SlPT4*, a late arbuscule developmental gene) (Liu et al., 2007; Buendia et al., 2016). Compared with control plants, the expression levels of all four genes were significantly reduced in VIGS-*SlLYK12*-infiltrated plants, suggesting that *SlLYK12* affects the development of AM symbiosis (**Figure 2H**).

### SlLYK1 Is Required for Chitin Responses

**FIGURE 3:**
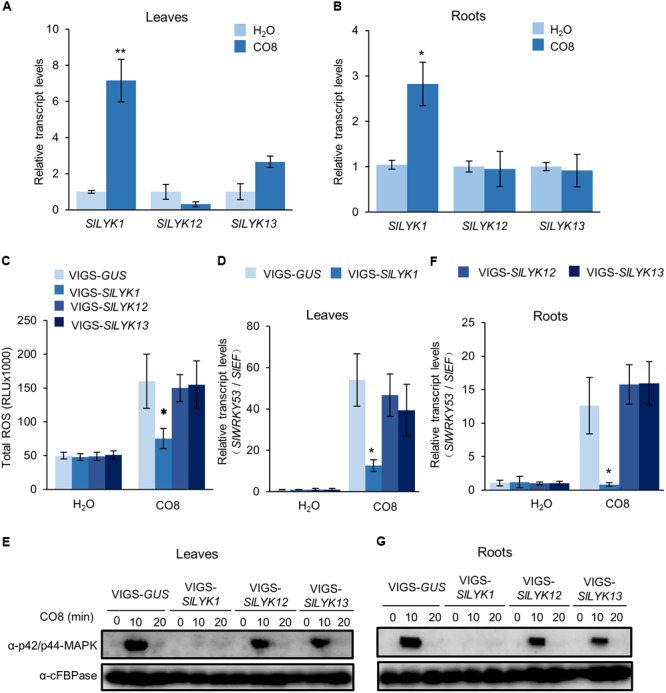
SlLYK1 is required for chitin responses. **(A,B)** Relative transcript levels of *SlLYK1, SlLYK12*, and *SlLYK13* after chitooctaose (CO8) treatment in leaves **(A)** and roots **(B)**. RNA was extracted from 2-week-old wild type leaves and roots 30 min after CO8 treatment. Transcript levels were detected by qRT-PCR. CO8-induced immune responses were analyzed in leaves **(C–E)** and roots **(F,G)** 4 weeks after infiltration with VIGS-*SlLYK1, -12, -13*, and VIGS*-GUS*. **(C)** CO8-induced reactive oxygen species (ROS) accumulation. ROS was measured using a chemiluminescence assay. Signals were recorded for 30 min and ROS were quantified as the total amount of light emitted (RLU). Data are expressed as means ± SD (*n* = 8). **(D,F)** CO8-induced *SlWRKY53* (*Solyc08g008280*) gene expression in leaves **(D)** and roots **(F)**. RNA was extracted 30 min after CO8 treatment, and gene expression was detected by qRT-PCR. Asterisks indicate significant differences from the VIGS-*GUS* control (Student’s *t*-test: ^∗^*P* ≤ 0.05, ^∗∗^*P* ≤ 0.01). **(E,G)** CO8-induced MAP kinase phosphorylation in leaves **(E)** and roots **(G)**. After CO8 treatment, MAP kinase phosphorylation was detected by immunoblot using the α-P42/P44 MAPK antibody and α-cFBPase (CYTOSOLIC FRUCTOSE-1,6-BISPHOSPHATASE) as a loading control. The experiment was repeated twice with similar results.

To determine whether *SlLYK1, SlLYK12*, and *SlLYK13* are required for chitin signaling, we analyzed their expression after CO8 treatment in 4-week-old leaves and roots. Our results indicated that *SlLYK1* gene expression is upregulated sevenfold after CO8 treatment in leaves and threefold in roots. *SlLYK13* is also slightly upregulated in leaves after CO8 treatment, whereas *SlLYK12* did not show significant upregulation in either leaves or roots (**Figures 3A,B**). These results suggest that *SlLYK12* might not have a role in chitin signaling. Next, we examined the responses of leaves infiltrated with VIGS-*SlLYK1, -SlLYK12, -SlLYK13*, and VIGS control to CO8 elicitation. First, ROS generation was measured after CO8 treatment, using a luminol-based chemiluminescence detection system. We found that VIGS-*SlLYK1*-inoculated leaves showed reduced ROS levels after CO8 treatment compared to the VIGS control, whereas plants infiltrated with VIGS-*SlLYK12* and *-SlLYK13* did not exhibit lower ROS levels (**Figure 3C**). Second, transcript levels of *SlWRKY53* (the ortholog of *AtWRKY53*), a typical chitin response gene (Cao et al., 2014), were detected by qRT-PCR. The results indicated that induction of *SlWRKY53* expression triggered by CO8 was significantly reduced in VIGS-*SlLYK1*-infiltrated plants (**Figure 3D**). Third, MAP kinase activity was analyzed by immunoblot assay. CO8 treatment causes MAP kinase phosphorylation, as demonstrated using an anti-p42/p44-MAPK antibody in VIGS control plants; however, levels of phosphorylation were significantly reduced in VIGS-*SlLYK1*-inoculated plants (**Figure 3E**). Similar to the results in leaves, CO8-triggered *SlWRKY53* gene expression and MAPK phosphorylation in roots was only reduced in VIGS-*SlLYK1* infiltrated plants (**Figures 3F,G**). Together, these results suggest that SlLYK1 plays a role in chitin signaling.

### SlLYK13 Is Involved in Cell Death

**FIGURE 4:**
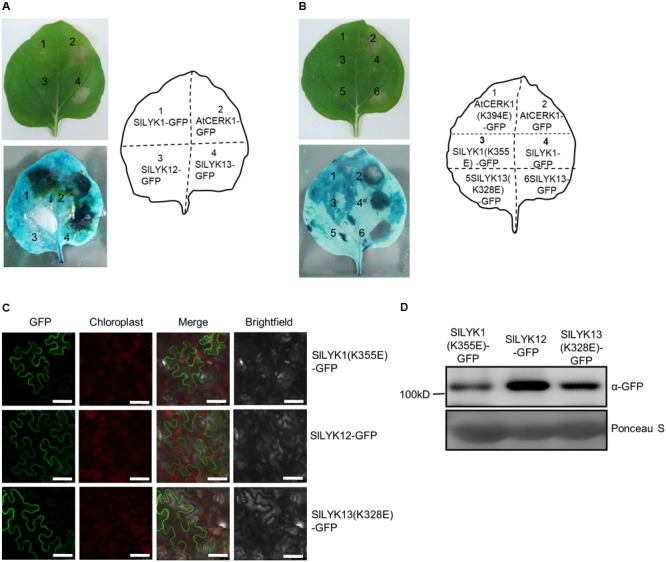
Overexpression of *SlLYK1* and *SlLYK13* induces cell death in *Nicotiana benthamiana* leaves. **(A)** Detection of cell death in *N. benthamiana* leaves. C terminus of AtCERK1, SlLYK1, SlLYK12, and SlLYK13 proteins were fused to green fluorescence protein (GFP) and then transiently expressed in *N. benthamiana*. Images were taken before (above) and after trypan blue staining (below) 3 days after infiltration. **(B)** Kinase inactivation of SlLYK1(K355E), SlLYK13(K328E), and AtCERK1(K394E) abolished the symptoms of cell death. **(C)** GFP observation. Images were taken using confocal microscopy 3 days after infiltration. Bars, 50 μM. **(D)** Size and abundance of the SlLYK1(K355E)-GFP, SlLYK12-GFP, and SlLYK13(K328E)-GFP proteins. Total proteins were extracted from leaves expressing the *SlLYK1(K355E)-GFP*, SlLYK*12-GFP*, and *SlLYK13(K328E)-GFP* constructs. Immunoblot analysis was performed using anti-GFP antibody. Ponceau S staining was used as a protein loading control. The experiment was repeated twice with similar results.

The results described above (sections “SlLYK12 Is Required for AM Symbiosis” and “SlLYK1 Is Required for Chitin Responses”) suggest that SlLYK1 is required for chitin signaling, while SlLYK12 is involved in AM symbiosis; hence, we wished to determine the function of SlLYK13. AtCERK1 has a chitin-independent role in cell death (Petutschnig et al., 2014), and ectopic overexpression of *AtCERK1* in *N. benthamiana* leaves results in symptoms of cell death (Pietraszewska-Bogiel et al., 2013); therefore, we analyzed the signs of cell death in *N. benthamiana* leaves ectopically overexpressing *SlLYK1, SlLYK12*, and *SlLYK13*. To this end, we fused the cDNA fragments encoding the proteins of interest to the 5′ of the *GREEN FLUORESCENT PROTEIN* (*GFP*) cDNA driven by the CaMV 35S promoter, respectively, and the resulting constructs were transiently expressed in *N. benthamiana* leaves using the same amount of agrobacteria. A positive control, overexpression of *AtCERK1-GFP*, caused leaf chlorosis and tissue collapse in the entire infiltrated region 3 days after agrobacterial infiltration. Compared with *AtCERK1-GFP*, overexpression of *SlLYK13-GFP* resulted in similar levels of cell death (**Figure 4A**). Overexpression of *SlLYK1-GFP* did not result in obvious tissue collapse; however, dead tissues (dark blue in color) were visible after trypan blue staining (**Figure 4A**). This symptom of cell death was never observed on overexpression of *SlLYK12-GFP* (**Figure 4A**).

We then examined whether SlLYK1- and SlLYK13-induced cell death were dependent on their kinase activities, by generating constructs with inactive kinase domains; *SlLYK1* (K355E) and *SlLYK13* (K328E). Our results showed that SlLYK1 and SlLYK13 lacking kinase activity did not cause symptoms of cell death (**Figure 4B**). All constructs were verified by confocal microscopy observation of the green fluorescence signal and immunoblot analysis to determine protein size (**Figures 4C,D**). Since tissue collapse leads to degradation of target proteins, SlLYK1- and SlLYK13-GFP proteins could not be detected by either method; however, SlLYK12-, SlLYK1(K355E)-, and SlLYK13(K328E)-GFP all showed green fluorescence signals in the cell periphery as expected for proteins predicted to localize in the plasma membrane (**Figure 4C**). Taken together, our results suggest that SlLYK13 and SlLYK1 have redundant functions in cell death, while overexpression of *SlLYK13* could cause more severe symptoms of cell death.

### SlLYK12 Is Subfunctionalized for AM Symbiosis in Tomato

**FIGURE 5:**
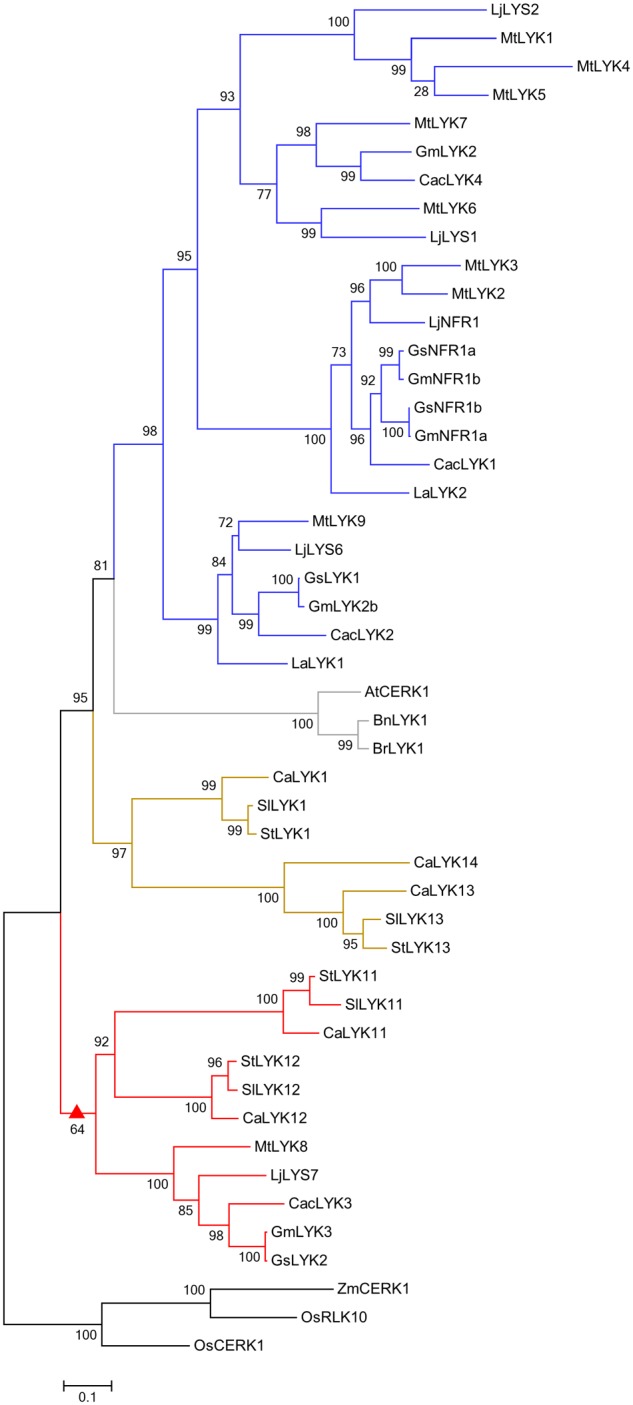
Phylogenetic tree of CERK1 homologs. An unrooted phylogenetic tree of CERK1 protein homologs was constructed using the approximately maximum-likelihood method. Species of origin: At, *Arabidopsis thaliana*; Bn, *Brassica napus*; Br, *Brassica rapa*; Ca, *Capsicum annuum*; Cac, *Cajanus cajan*; Gm, *Glycine max*; Gs, *Glycine soja*; La, *Lupinus angustifolius*; Lj, *Lotus japonicus*; Mt, *Medicago truncatula*; Os, *Oryza sativa*; Sl, *Solanum lycopersicum*; St, *Solanum tuberosum*; Zm, *Zea mays*. Branches are labeled with their respective bootstrap values. The solid triangle represents the common ancestor sub-functionalized for a role in AM symbiosis, the blue color represents the Leguminosae-specific subclade, the gray color represents the Cruciferae-specific subclade, the olive color represents the Solanaceae-specific subclade, and the red color represents the mixed subclade containing members from Leguminosae and Solanaceae.

To decipher the evolutionary relationships among LYK family proteins correlated with their functions in immunity and symbiosis, we constructed a phylogenetic tree by analyzing the protein sequences of CERK1 homologs from six Leguminosae species (*Glycine max, G. soja, Cajanus cajan, Lupinus angustifolius, M. truncatula*, and *L. japonicus*), three Solanaceae species (*Capsicum annuum, S. lycopersicum*, and *Solanum tuberosum*), three Cruciferae species (*A. thaliana, Brassica napus*, and *Brassica rapa*), and two Gramineae species (*Oryza sativa* and *Zea mays*). Finally, full length amino acid sequences encoded by 48 genes from 14 species were used to construct the phylogenetic tree (**Figure 5**).

Similar to other reported phylogenetic trees of LysM receptor proteins or kinases (Arrighi et al., 2006; Zhang et al., 2007, 2009; Lohmann et al., 2010; De Mita et al., 2014), CERK1 homologs from monocotyledonous and dicotyledonous species were assigned to two different groups (**Figure 5**). Monocotyledonous maize and rice only have one or two CERK1 paralogs, whereas dicotyledonous species other than the Cruciferae have evolved several homologs, suggesting that the *CERK1* family has experienced duplication events in dicotyledonous species during their evolution. The CERK1 homologs in Solanaceae and Leguminosae species are clustered into three clades: a Leguminosae-specific clade (blue), containing the Nod factor receptor NFR1/LYK3 and chitin receptor LjCERK6; a Solanaceae-specific clade (olive), including the immune receptors SlLYK1 and SlLYK13; and a mixed clade (red). SlLYK12, the potential Myc-factor receptor, was assigned to the mixed clade, which contains LYKs from both Solanaceae and Leguminosae species, suggesting that an ancestral gene duplication event occurred before the divergence of the Solanaceae and Leguminosae species. As OsCERK1 has a dual role in immunity and symbiosis, we hypothesize that an ancestor molecule in dicotyledons, which was responsible for both immunity and symbiosis, underwent gene duplication, leading to the sub-functionalization for a role in immunity and symbiosis, respectively.

## Discussion

In this study, we identified the orthologs of CERK1 in tomato, and investigated their function using a VIGS approach. Unlike rice OsCERK1, which has dual roles in chitin and AM symbiosis (Miyata et al., 2014; Zhang et al., 2015), we found that no single tomato CERK1 ortholog is responsible for both functions; rather, SlLYK1 mainly affects chitin signaling, while SlLYK12 is required for AM symbiosis. Therefore, we hypothesize that a gene duplication event and functional divergence occurred in an ancient ancestor of tomato. This subfunctionalization could be because of tissue-specific expression patterns. SlLYK12 is mainly expressed in the roots where AM symbiosis is established, whereas SlLYK1 showed equal expression levels in both leaves and roots. In addition, the expression of these genes was specifically induced by AM symbiosis and chitin, respectively.

Functional studies of LysM-RLP and LysM-RLK suggest that these receptors can function as hetero-oligomers (Kelly et al., 2017; Zipfel and Oldroyd, 2017; Desaki et al., 2018); for example, the hetero-oligomer of AtLYK5-AtCERK1 recognizes chitin in *Arabidopsis* (Cao et al., 2014), OsCEBiP-OsCERK1 recognizes chitin in rice (Shimizu et al., 2010; Hayafune et al., 2014), AtLYM1/3-AtCERK1 recognizes peptidoglycans in *Arabidopsis* (Willmann et al., 2011), OsLYP4/6-OsCERK1 recognizes peptidoglycan and chitin in rice (Liu et al., 2013; Ao et al., 2014), and LjNFR5-LjNFR1 recognizes Nod factor in *L. japonicus* (Broghammer et al., 2012). Therefore, it is very likely that SlLYK12 pairs with SlLYK10, the ortholog of NFR5 in tomato (Buendia et al., 2016). Plants with silenced SlLYK10 showed reduced AM colonization (Buendia et al., 2016). However, whether SlLYK10 associates with SlLYK12 awaits biochemical confirmation.

SlLYK1 and SlLYK13 were reported to have redundant roles in bacteria-mediated immune responses (Zeng et al., 2012). SlLYK1 (previously referred to as Bit9) and SlLYK13 both interact with the bacterial effector, avrPtoB, and plants with silenced *SlLYK1, SlLYK11, SlLYK12*, and *SlLYK13* were more susceptible to *Pseudomonas syringae* (Zeng et al., 2012). In this study, we found that ectopic expression of *SlLYK1* and *SlLYK13* in *N. benthamiana* induced cell death; however, SlLYK13 could trigger a stronger cell death response than SlLYK1. In addition, plants with silenced *SlLYK13* showed unaltered responses to chitin, suggesting that its involvement in cell death may be separate from chitin signaling. Indeed, in *Arabidopsis*, the cell death phenotype mediated by AtCERK1(L124F) is completely independent of chitin signaling (Petutschnig et al., 2014), but ectopic expression of *AtCERK1* in *N. benthamiana* strongly induces cell death. Therefore, tomato SlLYK1 and SlLYK13 may have undergone sub-functionalization after gene duplication during evolution.

CERK1 homologs in Solanaceae and Leguminosae species are clustered into three clades: a legume-specific clade (NFR1-MtLYK3-LjCERK6), a Solanaceae-specific clade (SlLYK1), and a mixed clade (SlLYK12-LjLYS7-MtLYK8). Consistent with the function of SlLYK12 in AM symbiosis, the SlLYK12-LjLYS7-MtLYK8 clade does not contain genes from *Lupinus angustifolius*, a legume species which cannot form AM symbiosis, but can establish a symbiotic relationship with rhizobia (Oba et al., 2001; Schulze et al., 2006), suggesting that the SlLYK12-LjLYS7-MtLYK8 clade may mediate the host specificity of AM symbiosis. In this clade, LjLYS7 and MtLYK8 are the closest orthologs of SlLYK12 in *L. japonicus* and *M. truncatula*, which were also predicted to be involved in symbiotic perception in endomycorrhizae in other phylogenetic analysis (De Mita et al., 2014). Given the common pathways used for AM symbiosis and legume-rhizobium symbiosis, it has been hypothesized that Nod factor receptors evolved from a Myc factor receptor (Lohmann et al., 2010; De Mita et al., 2014). According to this theory, it is reasonable to predict that the putative Myc factor receptor should be clustered with NFR1 in a single group. Surprisingly, we found that the NFR1 clade is more closely related to the SlLYK1-immunity clade than to the SlLYK12-AM symbiosis clade. Consistent with this notion, LjCERK6, the closest paralog of NFR1, was recently identified as involved in chitin recognition in *L. japonicus*, but not AM symbiosis (Bozsoki et al., 2017). All these studies suggest that NFR1 might not have evolved directly from a receptor for AM symbiosis, rather, it may have evolved from an ancestor with a dual function, which underwent gene duplication in legumes and the paralogous gene underwent neofunctionalization to become a Nod factor receptor. Alternatively, it is interesting to note that the YAQ motif that is proposed to be associated with a role in symbiosis in the CERK1 family is conserved in SlLYK1 and SlLYK12 (Nakagawa et al., 2011; De Mita et al., 2014), so it is possible that SlLYK1 might have a symbiotic role. However, this role might not have been observed because of redundancy with SlLYK12 or because of incomplete silencing. Indeed, PsLYK9, the ortholog of LjCERK6 in *Pisum sativum*, is required for plant immunity and could be involved in Myc factor perception (Leppyanen et al., 2018). Therefore, the evolutionary origin of Nod factor receptors awaits future experimental investigation.

## Author Contributions

YL designed the research and wrote the manuscript. DL and XS performed most of the experiments. NW contributed to analysis of gene expression. FS designed primers for VIGS constructs and provided technique support for VIGS approach.

## Conflict of Interest Statement

The authors declare that the research was conducted in the absence of any commercial or financial relationships that could be construed as a potential conflict of interest.

## Footnotes

1 http://www.microbesonline.org/fasttree/
