# Supplementary material for: Tomato LysM Receptor-Like Kinase SlLYK12 Is Involved in Arbuscular Mycorrhizal Symbiosis
Source: Front Plant Sci. 2018 Jul 11;9:1004. doi: 10.3389/fpls.2018.01004 (PMC6050466; doi:10.3389/fpls.2018.01004)
Supplement: Supplementary file 1 [file Data_Sheet_1.DOCX]

Supplementary Material

**Tomato LysM RECEPTOR-LIKE KINASE (SlLYK12) is involved in arbuscular mycorrhizal symbiosis**

Dehua Liao ^#¶^, Xun Sun^#^, Ning Wang, Fengming Song, Yan Liang^*^

College of Agriculture and Biotechnology, Zhejiang University, Hangzhou, 310058, China

*** Correspondence:**Yan Liang

[yanliang@zju.edu.cn](mailto:yanliang@zju.edu.cn)

**^#^** These authors contributed equally

^¶^ Present address: FAFU-UCR Joint Center for Horticultural Plant Biology and Metabolomics, Haixia Institute of Science and Technology, Fujian Agriculture and Forestry University, Fuzhou, Fujian 350002, China


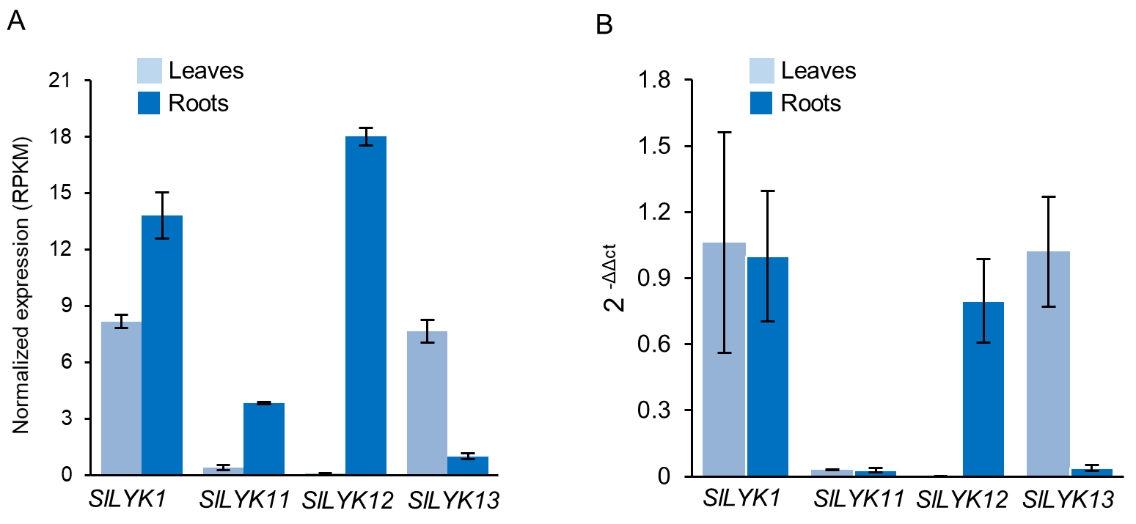


**Supplementary Figure 1.** **The transcript levels of *SlLYK1, SlLYK11, SlLYK12* and *SlLYK13*. (A)** Previous RNA sequencing data of *SlLYK1, SlLYK11, SlLYK12* and *SlLYK13* ([Tomato Genome, 2012](#_ENREF_1))*.* (B) The transcript levels of *SlLYK1, SlLYK11, SlLYK12* and *SlLYK13* in leaves and roots in our condition. RNA was extracted from two-week-old leaves and roots, and the gene expression levels were detected by qRT-PCR. Data are mean ± SD from three biological replicates.

**Table S1: primer sequences used in this study**

| Name | Sequence |
| --- | --- |
| SlEF-qRT-F | GGAACTTGAGAAGGAGCCTAAG |
| SlEF-qRT-R | CAACACCAACAGCAACAGTCT |
| SlLYK1-qRT-F | TTCTATGGAAATCAAGGC |
| SlLYK1-qRT-R | TTACACTATTCAAGCAAG |
| SlLYK11-qRT-F | ATTGAGACCAAGTATGAG |
| SlLYK11-qRT-R | AGTGTAACTTGTGCTTCT |
| SlLYK12-qRT-F | TGAGGTCCATTGTTGTAG |
| SlLYK12-qRT-R | GTTGAAAATGCTTGAGTG |
| SlLYK13-qRT-F | AAATCAGTAGTGGTGGCT |
| SlLYK13-qRT-R | CTATTACTTTGAGGAGGC |
| SlPT4-qRT-F | ATGGCGGGCAGAATGAGA |
| SlPT4-qRT-R | GCACAAGGCGTAGTGATG |
| SlBCP -qRT-F | GACCGGCTACTAAGGATGATTC |
| SlBCP-qRT-R | CTCTTGAAGTAGGCAAAGCTCTA |
| SlRAM2-qRT-F | AAGCCAGGAGTACTTGTTGGTAA |
| SlRAM2-qRT-R | TGGACTAAACGACCATCATGGA |
| RiGAPDH-qRT-F | GACGTCTCAGTTGTTGATTTA |
| RiGAPDH-qRT-R | TTTGGCATCAAAAATACTAGA |
| WRKY53-qRT-F | CCACAACCAACATCGCCAGAGAA |
| WRKY53-qRT-R | ACGGTGAATAGCCGCTACCTATCA |
| SlLYK1-vigs-F | GGGGACAAGTTTGTACAAAAAAGCAGGCTACAAATCAAGGCATGATAAAC |
| SlLYK1-vigs -R | GGGGACCACTTTGTACAAGAAAGCTGGGTCATGTTCCATTTCCAACTC |
| SlLYK12-vigs -F | GGGGACAAGTTTGTACAAAAAAGCAGGCTACATGAATATCAGAGCGAAAA |
| SlLYK12-vigs -R | GGGGACCACTTTGTACAAGAAAGCTGGGTCCAATCACAAGAAAATGGG |
| SlLYK13-vigs -f | GGGGACAAGTTTGTACAAAAAAGCAGGCTACATGATATTCCTAAGAAGGAG |
| SlLYK13-vigs -r | GGGGACCACTTTGTACAAGAAAGCTGGGTCAAATACATGGCCCAGAAACT |
| SlLYK1-F | GGGGACAAGTTTGTACAAAAAAGCAGGCTAcATGTTTGAATCCAGGCCAAG |
| SlLYK1-R | GGGGACCACTTTGTACAAGAAAGCTGGGTCCCTTCCAGACATGAGGTTTA |
| SlLYK12-F | GGGGACAAGTTTGTACAAAAAAGCAGGCTAcATGAATATCAGAGCGAAAAC |
| SlLYK12-R | GGGGACCACTTTGTACAAGAAAGCTGGGTCCCTTCCTGACATGAGATGGT |
| SlLYK13-F | GGGGACAAGTTTGTACAAAAAAGCAGGCTACATGATATTCCTAAGAAGGAG |
| SlLYK13-R | GGGGACCACTTTGTACAAGAAAGCTGGGTCGGCATGTGACGATGAAAGTG |
| SlLYK1(K355E)-F | GAGAAAGCAGCCATCGAGAAAATGGACATGG |
| SlLYK1(K355E)-R | CCATGTCCATTTTCTCGATGGCTGCTTTCTC |
| SlLYK13(K328E)-F | AAGAAAGCAGCAATTGAGCGAATGAACAGGG |
| SlLYK13(K328E)-R | CCCTGTTCATTCGCTCAATTGCTGCTTTCTT |

**Table S2: The amino acid sequences of *LYK* genes for phylogenetic analysis**

| **Gene name** | **Accession number** | **Sequence** |
| --- | --- | --- |
| AtCERK1 | NP_566689.2 | MKLKISLIAPILLLFSFFFAVESKCRTSCPLALASYYLENGTTLSVINQNLNSSIAPYDQINFDPILRYNSNIKDKDRIQMGSRVLVPFPCECQPGDFLGHNFSYSVRQEDTYERVAISNYANLTTMESLQARNPFPATNIPLSATLNVLVNCSCGDESVSKDFGLFVTYPLRPEDSLSSIARSSGVSADILQRYNPGVNFNSGNGIVYVPGRDPNGAFPPFKSSKQDGVGAGVIAGIVIGVIVALLLILFIVYYAYRKNKSKGDSFSSSIPLSTKADHASSTSLQSGGLGGAGVSPGIAAISVDKSVEFSLEELAKATDNFNLSFKIGQGGFGAVYYAELRGEKAAIKKMDMEASKQFLAELKVLTRVHHVNLVRLIGYCVEGSLFLVYEYVENGNLGQHLHGSGREPLPWTKRVQIALDSARGLEYIHEHTVPVYVHRDIKSANILIDQKFRAKVADFGLTKLTEVGGSATRGAMGTFGYMAPETVYGEVSAKVDVYAFGVVLYELISAKGAVVKMTEAVGEFRGLVGVFEESFKETDKEEALRKIIDPRLGDSYPFDSVYKMAELGKACTQENAQLRPSMRYIVVALSTLFSSTGNWDVGNFQNEDLVSLMSGR |
| SlLYK12 | NP_001234725.1 | MNIRAKTINLSSFFLFIILNGEAKSCGNGCEMAIASYHIWSGANLTYISHLFNLTIPVILNYNPQITNQDSITSDTRINLPFSCDCLNGDFLGHTFVYKTVFGDTYKKVATMAFANLTTEYWLKRVNNYDPTSIPDYAMINVTVNCSCGDGEVSDDYGLFATYPIRPGENLSTVAVGSGVPAELLQKFNPGLDFGSGSGIVFVPARDAHGNFPPLKTRSRGLSRGAIAGTTVAAIFGATFFVVCVYFVFYRSKQAEEESFLQGSSDEHFNENFRPPNLEKITESGPLFGVISPRPTGITVDKSVEFSYEELAKATNNFSMENKIGQGGFGLVFYGMLKGERAAIKKMDMQASKEFFAELKVLTHVHHLNLVRLIGYCVEGSLFLVYEYIENGNLGEHLRGSSRNPLSWSTRVQIALDAARGLEYIHEHTVPLYIHRDIKSANILIDKDFRAKVADFGLTKLTEVGSTSFHTRLVGTFGYMPPEYAQYGDVSPKVDVYAFGVVLYELISAKEAIVKTNEVITESKGLVALFEDVLHQSGGAREGLCKVVDPKLGDDYPLDSVCKVAQLAKACTHENPQLRPSMRSIVVALMTLSSSTEDWDIGSFYENHLMSGR |
| SlLYK1 | NP_001233773.1 | MFESRPRSVLSLGVFVILVYLSSVPLPVNSQCNRGCDLALASFYVWRGSNLTLISEMFSTSIADIVSYNNRDNIPNQDSVIAGTRINIPFRCDCLNDGEVLGHAFPYRVKSGDTYDLVARNYSDLTTAQWMMKFNSYPENNIPNTVNLSVVVNCSCGNSDVSKDFGLFVTYPVRAEDNLTSVASAANVSEDIIRRYNPAAVSILDIGQGIIYIPGRDRNGNFPPLPTSTDGLSGGAKAGISIGAIGVVLLLAGLVYVGCYRNKTRKISLLRSEDHLHQYGHGPEGSTTVKAADSGRLADGNSPVLSGITVDKSVEFTYEELATATNDFSIANKIGQGGFGAVYYAELRGEKAAIKKMDMEATREFLAELKVLTNVHHLNLVRLIGYCVEGSLFLVYEYVENGHIGQHLRGTGRDPLPWSKRVQIALDSARGLEYIHEHTVPVYIHRDIKTANILIDKNFHAKVADFGLTKLTEVGSSSLQTRLVGTFGYMPPEYAQYGDVSPKVDVYAFGVVLYELISAKEAIVKPNGSVTESKGLVALFEEVLNQPDPDEDLRQLVDPRLGDDYPLDSVRKMAQLAKACTHENPLIRPSMRSIVVALMTLSSSTEDWDVGSFYGNQGMINLMSGR |
| SlLYK13 | NP_001234730.1 | MIFLRRRSITILVLIYFFSNCTTCYSTSCTNGCDLALASFFIWPESNLPLINQLFDNISYSDILEWNTQITSTFILTESRVHVPFRCDCLNNGEFLGHVFSYNVSANETYDLIATRRYSSLTNKELLMRDNRYPDNNIPDHVTLNVTVNCSCGNKHVSKDYGLFITYPMRPGENLSYIALVTNTSSKLIEMYNPMVNFSAGSGLLYIPGRDKLGNYPPISTRKGSSGKTIAALAVASLAGVLLLVGIIYVGIYRRKEQKVAANIPVSSGQCYPPSPGLSGIHVDKSVEFSYQELAESTDNFSSNKIGEGGFGAVYYAELRGKKAAIKRMNRERTEFLAELKILTRVHHLNLVSLIGYCVERSLFLVYEFIENGNLSQHLHGRDVLTWSTRVQIAMDSARGLEYIHEHTVPFYIHRDVKSANILINKNFHAKIGDFGLSKLVESGNPTLNTRFMGTFGYMPPEYGHSGVISRKVDVYAFGVVLYELISSKDAIVKEDGVDEARSLVALFDEAHSHPNQIEAISRLIDPKLCDDYPLDSVKMAQLAKSCTEKNPEMRPTMKSVVVALMALSSHA |
| SlLYK11 | NP_001234719.1 | MIFFQENRSFELVLGLLVLNILWVGVKSQCSDDCDALASFYVWNGANLTFMSNTFSTPIKNILSYNPQITNPDIIQSQSRVNVPFSCSCVDGKFMGHQFDVQVKTNTTYPRITRLYCSNLTTVEKLQESNSYDPNNVPVNSIVKVIVNCSCGNSHVSKDYGLFITYPLRPGENLVTLANDFSLPQKLLEDYNPEANFSSGSGLVFIPGKAQKVSLILLLQRHCFQLIKSEVSKSSECDCNLLKTMLLTGFSGGAITGISVAVVLVVALLAVCIYITFYRGRKTEENLNLEPYKHSSNKHIPGHANFENSSEGGSLKQGASPEVPRIAVDKSIEFSYDELAKASDNFSTAYKIGQGGFASVYYGELRGEKAAIKKMDMQATKEFLAELKVLTHVHHLNLVRLIGYCVEGSLFLVYEYIENGNLSQHLRGFVPGKVPLPWSTRVKIALDAARGLEYIHEHTVPVYIHRDIKTANILIDKNFRAKVADFGLTKLIETEGGSMNTRLVGTFGYMAPEYGQFGNVSLKIDVYAFGVVLYELISARKAIIKTSEISTESKGLVGLFEDVLNEVDPKEGICKLVDPKLGDDYPLDSVWNVALLAKACTQENPQLRPSMRSIVVALMTISSTSTADWNLGEFYENQGLAHLISGR |
| MtLYK1 | AAQ73154.1 | MKPIKFILSLLLMLLASSSAESKCSKTCDLALASYYIWEGTNLTYISNIMQSNVVSKPLDIFSYNTDTLPNLDMLRFSSRLNVPFPCDCINDEFLGHTFLYEFHPRETYASIAELTFSNLTNKEWMEKVNVPDSVKVNVTVNCSCGDKMVSKDYGLFITYPLSSEDTLESIAKHTKVKPELLQKYTPGVNFSKGSGLVFIPGKDKNGVYVPLPHGKAGHLARSLATAVGGTCTVLLLAISIYAIYFRNKNAKESKLPSKYIVVDKSPKFSYEELANATDKFSLANKIGQGGFGEVYYGEPRGKKTAIKKMKMQATRELAELKILTRVHHCNLVHLIGYCVEGSLFLVYEYIDNGNLSQNLHDSERGPMTWSTRMQIALDVARGLEYIHEHSVPVYIHRDIKSDNILLNENFTGKIADFGLTRLTDSANSTDNTLHVAGTFGYMPPENVYGRISRKIDVYAFGVVLYELISAKPAVIKIDKTEFESEIRTNESIDEYKSLVALFDEVIDQKGDPIEGLRNLVDPRLEDNYSIDSISKMAKLARACLNRDPKRRPTMRAVVVSLMTLNSTIDDGSRSASAALSTVMEHDSK |
| MtLYK2 | CAN88845.1 | MKLKNGLLLFFMFLECVFSKVESKCVKGCDVALASYHVMLPFTYQNITSFMQSKIVSVSSLSDVIISYNKGKVSKNGNLFAFSRVNIPFPCECIGGDFLGHVFEYSAKEGDTYDLIANSYYASLTTVELLKKFNSYDQDHIPAKAKVNVTVNCSCGNSQISKDYGLFITYPLRTDDTLQKIANQSNLDEGLIQSYNSGVNFSNGSGIVFIPGRDQNGDYVPLYPRSGLAKGATVGIIIAGIFGLLLLVIYIYVRYFKKKEEEKTKLAEALSTQDGSAEYETSGSSVHATVFTGIMVAKSTEFSYQELAKATNNFSLDNKIGQGGFGAVYYAELRGEKTAIKKMDVQASSEFLCELKVLTHVHHLNLVRLIGYCVEGSLFLVYEHIDNGNLGQYLHGTGKEPLPWSSRVEIALDSARGLEYIHEHTVPMYIHRDVKSANILIDKNLRGKVADFGLTKLLEVGNSTLQTRLVGTFGYMPPEYAQYGDVSPKIDVYAFGVVLFELISAKNAVLKTGEFVAESRGLVALFEEALNQTDPLESLRKLVDPRLREDYPIDSVLKMAQLGRECTKDNPLLRPSMRSIVVSLMSLLSPSEDCDGDTSDENQTIINLLSVR |
| MtLYK3 | XP_003616958.1 | MNLKNGLLLFILFLDCVFFKVESKCVKGCDVALASYYIIPSIQLRNISNFMQSKIVLTNSFDVIMSYNRDVVFDKSGLISYTRINVPFPCECIGGEFLGHVFEYTTKEGDDYDLIANTYYASLTTVELLKKFNSYDPNHIPVKAKINVTVICSCGNSQISKDYGLFVTYPLRSDDTLAKIATKAGLDEGLIQNFNQDANFSIGSGIVFIPGRDQNGHFFPLYSRTGIAKGSAVGIAMAGIFGLLLFVIYIYAKYFQKKEEEKTKLPQTSRAFSTQDASGSAEYETSGSSGHATGSAAGLTGIMVAKSTEFTYQELAKATNNFSLDNKIGQGGFGAVYYAELRGEKTAIKKMDVQASSEFLCELKVLTHVHHLNLVRLIGYCVEGSLFLVYEHIDNGNLGQYLHGIGTEPLPWSSRVQIALDSARGLEYIHEHTVPVYIHRDVKSANILIDKNLRGKVADFGLTKLIEVGNSTLHTRLVGTFGYMPPEYAQYGDVSPKIDVYAFGVVLYELITAKNAVLKTGESVAESKGLVQLFEEALHRMDPLEGLRKLVDPRLKENYPIDSVLKMAQLGRACTRDNPLLRPSMRSIVVALMTLSSPTEDCDDDSSYENQSLINLLSTR |
| MtLYK4 | XP_003616957.2 | MNLKNGLLLFILFLDCVFFKVETKCVKGCDVALASYYIMPSIQLINVSNFIQSKIVLTNSFDVIMSYNRVVVFDKSGLISYTRINVPFPCECIGGEFLGHVFEYTTKEGDDYDLIANTYYASLTTVELLKKFNSYDPNHIPVKAKINVTVICSCGNSQISKDFGLFVTYPLRSDDTLAKIATKADLDEGLLQNFNQDANFSKGSGIVFIPGRDENGVYVPLPSRSLVAAGICIRGVCMVLLLAICIYVRYFRKKNGEESKLPPEDSMSPSTKDGDKDSYSDTRSKYILVDKSPKFSYKVLANATENFSLAKKIGQGGFGEVYYGVLGGKKVAIKKMKTQATREFLSELKVLTSVRHLNLVHLIGYCVEGFLFLVYEYMENGNLSQHLHNSEKELMTLSRRMKIALDVARGLEYIHDHSVPVYIHRDIKSDNILLNKNFNGKIADFGLTKLTNIANSTDNTNHMAGTFGYMPPENAYGRISRKMDVYAFGVVLYELISAKAAVIMIDKNEFESHEIKTNESTDEYKSLVALFDEVMDQKGDPIEGLRKLVDPRLGDNYSIDSISKMAKLAKACINRDPKQRPKMRDVVVSLMKLISTIDDESRTDSAELSLDVEHDSN |
| MtLYK5 | CAN88846.1 | MEQPLKFRLSLLFLLLVLQSITSESKCSKTCDLALASYYIRPGTTLANISKVMQSNVVSKEEDILSYNTAITNIDAIQSDTRVNVPFPCDCINDEFLGHTFLYKLRLGDIYPSIAERTYTNLTTEEWMERVNSYPGTDLPVSAMVNVTVNCSCGSREVSKDYGLFITYPLSSKDTLESISKDTMIEAELLQRYNPGVNFSQGSGLVFIPGKDENGFYVPLPPRKGHLARSLGTAGISIGGLCMVLLLLLCIYVRYFRMKNGEEKSKLSPDDSMTPSTKDVDKDTNGDTGSRYIWLDKSPEFSYEELANATDNFSLAKKIGQGGFGEVYYGELRGQKIAIKKMKMQATREFLSELKVLTSVHHRNLVHLIGYCVEGFLFLVYEYMENGNLNQHLHNSEKEPITLSTRMKIALDVARGLEYIHDHSIPVYIHRDIKSDNILLNENFTGKVADFGLTKLTDAASSADNTDHVAGTFGYMPPENAYGRISRKIDVYAFGVVLYELISAKAAVIKIDKTEFELKSLEIKTNESIDEYKSLVALFDEVMDQTGDPIEGLRKLVDPRLGYNYSIDSISKMAKLAKACINRDPKQRPKMRDLVVSLMKLNYTIDDESRTGSAELSLAVEHDSN |
| MtLYK6 | AAQ73157.1 | MKISFSFIVLILLIASTESKCNEGCSLALASYTLNHVSNLTYISNIMKSNVLSKPQDIIINNDKNKRANVPFPCNCINGEFLAYTFLYELQPGETYTSVAEESFSNLTTDVWMQNFNVYRPTNIPDFAMIKVTVNCSCGNKEVSMDYGLFITYPLRSEDTLESIAKGAEIEAELLQRYNPGVNFSKGSGLVFIPGKDQNGSYLPLHPSTVGTVAITGISVGVLAALLLLLFFVYIKYYLKKKNKKTWEKNLILDDSKMKSAQIGTNIASIMVEKSEEFSYKELSIATNNFSMANKIGEGGFGEVFYAELRGQKAAIKKMKMKASKEFCAELKVLTLVHHLNLVGLIGYCVEGFLFLVYEYIDNGNLSQNLHDSEREPLSWSTRMQIALDSARGLEYIHEHTVPVYIHRDIKSENILLDKSFCAKVADFGLSKLADVGNSTSSTIVAEGTFGYMPPEYACGSVSSSPKVDVYAFGVVLYELISAKAAFDEVFGYDQDPTEGIKNLVDPRLGDNYSIDSVCKMAQLAKACTMRDPQLRPSMRSIVVALMTLTSTTEDWNISSFYENPAFLNLMSGK |
| MtLYK7 | AAQ73158.1 | MKPIKFRLSFLFMLLASKSFIAESKCSKTCNIALASYYLQDDTNLTYVSNIMQSNLVTKPEDIVSYNTDTITNKDFVQSFTRVNVPFPCDCIHDEFLGHIFQYQVATKDTYLSVASNNYSNLTTSEWLQNFNSYPSNDIPDTGTLNVTVNCSCGNSDVSKDYGLFITYPLRPEDSLELISNKTEIDAELLQKYNPGVNFSQGSGLVYIPGKDQNRNYVPFHISTGGLSGGVITGISVGAVAGLILLSFCIYVTYYRKKKIRKQEFLSEESSAIFGQVKNDEVSGNATYGTSDSASPANMIGIRVEKSGEFSYEELANATNNFNMANKIGQGGFGEVYYAELNGEKAAIKKMDMKATKEFLAELKVLTRVHHVNLVRLIGYCVEGSLFLVYEYIDNGNLGQHLRSSDGEPLSWSIRVKIALDSARGLEYIHEHTVPTYIHRDIKSENILLDKNFCAKVADFGLTKLIDAGISSVPTVNMAGTFGYMPPEYAYGSVSSKIDVYAFGVVLYELISAKAAVIMGEDSGADLKGLVVLFEEVFDQPHPIEGLKKLVDPRLGDNYPIDHVFKMAQLAKVCTNSDPQQRPNMSSVVVALTTLTSTTEDWDITSIFKNPNLVNLMSGR |
| MtLYK9 | XP_003601376.2 | MEHQPRFTSFISLPLFSIFLASIPFITESKCTKGCSLALANFYVSQGSNLTYISSIMRSNIQTRPEDIVEYSREIIPSKDSVQAGQRLNVPFPCDCIDGQFLGHKFSYDVETGDTYETVATNNYANLTNVEWLRRFNTYPPNDIPDTGTLNVTVNCSCGDADVGNYALFVTYPLRPGETLVSVANSSKVDSSLLQRYNPGVNFNQGSGIVFVPGKDQNGSFVFLGSSSGLGGGAIGGIAVGIVVVLLLVAAAIYFGYFRKKKIQKEELFSRDSTALFSQDGKDENSHGAANVTQRPGVMTGITVDKSVEFSYDELAAASDNFSMANKIGQGGFGSVYYAELRGEKAAIKKMDMQATKEFLAELKVLTRVHHLNLVRLIGYSIEGSLFLVYEYIENGNLSQHLRGSGRDPLPWATRVQIALDSARGLEYIHEHTVPVYIHRDIKPANILIDKNFRGKVADFGLTKLTEVGSSSLPTGRLVGTFGYMPPEYAQYGDVSPKVDVYAFGVVLYELISAKEAIVKSSESVADSKGLVGLFEGVLSQPDPTEDLRKIVDPRLGDNYPADSVRKMAQLAKACTQENPQLRPSMRSIVVALMTLSSTTDDWDVGSFYENQNLVNLMSGR |
| OsCERK1 | XP_015650771.1 | MEASTSLLVLVLAAAAFAAGTVTEAAGDGCSAGCDLALASFYVTPNQNVTNMADLFGIGAANYRSLAPYNPNIPNLDFINVGGRVNVYFTCGCRSLPGSPGATYLAGAFPFQMSRGQIYTSVAANYNNLTTAEWLQATNSYPANNIPDTAVINATVNCSCGDASISPDYGLFLTYPLRAEDTLASVAATYGLSSQLDVVRRYNPGMESATGSGIVYIPVKDPNGSYLPLKSPGKGASAGAIAGGVVAGVVVLAAIFLYIIFYRRRKAKQATLLQSSEDSTQLGTISMDKVTPSTIVGPSPVAGITVDKSVEFSYEELSNATQGFSIGNKIGQGGFGAVYYAELRGEKAAIKKMDMQATHEFLAELKVLTHVHHLNLVRLIGYCIESSLFLVYEFIENGNLSQHLRGMGYEPLSWAARIQIALDSARGLEYIHEHTVPVYIHRDIKSANILIDKNYRAKVADFGLTKLTEVGGTSMPTGTRVVGTFGYMPPEYARYGDVSPKVDVYAFGVVLYELISAKEAIVRSTESSSDSKGLVYLFEEALNSPDPKEGLRTLIDPKLGEDYPIDSILKLTQLAKVCTQEDPKLRPSMRSVVVALMTLSSTSEFWDMNNLYENQGLVNLMSGR |
| OsRLK10 | XP_015611968.1 | MFSLPALLIGACAFAAAAVAASGDGCRAGCSLAIAAYYFSEGSNLTFIATIFAIGGGGYQALLPYNPAITNPDYVVTGDRVLVPFPCSCLGLPAAPASTFLAGAIPYPLPLPRGGGDTYDAVAANYADLTTAAWLEATNAYPPGRIPGGDGRVNVTINCSCGDERVSPRYGLFLTYPLWDGETLESVAAQYGFSSPAEMELIRRYNPGMGGVSGKGIVFIPVKDPNGSYHPLKSGGMGNSLSGGAIAGIVIACIAIFIVAIWLIIMFYRWQKFRKATSRPSPEETSHLDDASQAEGIKVERSIEFSYEEIFNATQGFSMEHKIGQGGFGSVYYAELRGEKTAIKKMGMQATQEFLAELKVLTHVHHLNLVRLIGYCVENCLFLVYEFIDNGNLSQHLQRTGYAPLSWATRVQIALDSARGLEYLHEHVVPVYVHRDIKSANILLDKDFRAKIADFGLAKLTEVGSMSQSLSTRVAGTFGYMPPEARYGEVSPKVDVYAFGVVLYELLSAKQAIVRSSESVSESKGLVFLFEEALSAPNPTEALDELIDPSLQGDYPVDSALKIASLAKSCTHEEPGMRPTMRSVVVALMALTANTDLRDMDYHPF |
| StLYK12 | XP_006348156.1 | MNIRAKTINLTISFFLFILNGEAKSCGNGCDMAIASYHIWSGANLTYISHIFNLTIPVILNYNPQITSQDSITSDTRLNLPFSCDCLNGDFLGHTFMYKTVFGDTYKKVATMAFANLTAEYWLKRVNNYDPTNIPNYAMINVTVNCSCGDGEVSDDYGLFATYPIRPGENLSTVAVGSGVSAELLEKFNPGLDFGSGSGIVFVPARDAQGNFPPLKTRSRGLSRGAIAGITVAAIFGATFFVVCVYFVFYRSKQIEEESFLQGSSDEHFNENFRPPNLEKITESGPLFGVISPRPTGITVDKSVEFSYEELAKATDNFSMENKIGQGGFGLVFYGMLKGERAAIKKMDMQASKEFFAELKVLTHVHHLNLVRLIGYCVEGSLFLVYEYIENGNLGEHLRGSSRNPLSWSTRVQIALDAARGLEYIHEHTVPLYIHRDIKSANILIDKDFRAKVADFGLTKLTEVGSTSFHTRLVGTFGYMPPEYAQYGDVSPKVDVYAFGVVLYELISAKEAIVKTNEVITESKGLVALFEDVLHQSDGAREGLCKVVDPKLGDDYPLDSVCKVAQLAKACTHENPQLRPSMRSIVVALMTLSSSTEDWDIGSFYENQGLVHLMSGR |
| StLYK1 | XP_006357945.1 | MFESRPRSVLSLGVFVILVYLSSVPLPVNSQCNRGCDLALASFYVWRGSNLTLISEMFSTSIPDIVSYNNRGNIPNQDSVIAGTRINIPFRCDCLNDGEVLGHAFPYRIKTGDTYDLVAKNYSDLTTAQWMMKFNSYPENDIPNTGNLSVVVNCSCGNGDVSKDFGLFVTYPVRAEDNLTSVASAANVSEDIIRRYNPAAESILDIGQGIIYIPGRDRNGNFPPLPTSTDGLSGGAKAGISIGAIGVVLLLAGLVYVGCYRNKTQKVSLLRSEDHLHQYGHGPEGSMTVKAADSGRLADGNSPGLSGITVDKSVEFTYEELATATNDFSIANKIGQGGFGAVYYAELRGEKAAIKKMDMEATREFLAELKVLTNVHHLNLVRLIGYCVEGSLFLVYEYVENGHIGQHLRGAGRDPLPWSKRVQIALDSARGLEYIHEHTVPVYIHRDIKTANILIDKNFHAKVADFGLTKLTEVGSSSLQTRLVGTFGYMPPEYAQYGDVSPKVDVYAFGVVLYELISAKEAIVKPNGSVTESKGLVALFEEVLNQPEPDEDLRQLIDPRLGDDYPLDSVRKMAQLAKACTHENPLIRPSMRSIVVALMTLSSSTEDWDVGSFYGNQGMINLMSGR |
| StLYK11 | XP_006348155.1 | MIFQENRSFELVLGLLVLNILWVGAKSQCSDDCDALASFYVWNGANLTFISNTFSTTIKHILSYNPQITNPDIIQFQSRVNVPFSCSCVDGKFMGHQFDVQVKTSTTYPRIARLYCSNLTTVEKLQESNSYDPNNVPVNAIVKVTVNCSCGNSHVSKDYGLFITYPLRPGENLVTVANDFNLPQKLLEDYNPEANFSRGSGLVFIPGKDQNGTYPPLRTSTSSKGFSGGAITGISVAAVLVVALLAVCIYITFYRGRKTEENLNLEPYKHSSNKHVPGQANFENSSEGGSLNKGASPEIPRIAVDKSVEFSYDELANASDNFSTAYKIGQGGFASVYYGELRGEKAAIKKMDMQATKEFLAELKVLTHVHHLNLVRLIGYCIEGSLFLVYEYIENGNLSQHLRGFVPGKVPLPWSTRVKIALDAARGLEYIHEHTVPVYIHRDIKTANILIDKNFRAKVADFGLTKLIETEGGSMNTRLVGTFGYMAPEYGQFGDVSPKIDVYAFGVVLYELISAKQAIIRNSEIATESKGLIGLFEDVLNEVDPREGICKLVDPKLGDDYPLDSVWNVALLAKACTQENPQLRPSMRSIVVALMTISSTSADWNLGEFYENQGLAHLISGR |
| StLYK13 | XP_006361893.1 | MIFLRRRSIIILVLIYLFSNCTTCSCINGCDLALASLFIWPESDLPKISQLFDNMPSNDILDWNTEITSTFILTESRVNVPFRCDCLNNGEFLGHVFSYIVSANDTYDLIATRLYSSLTNKELLMRDNSYPENNIPVHVTLNVTVNCSCGNKDVSKDYGLFITYPMRPGENLSYIALVTNTSSRLIEMYNPMVNFSAGSGLLYIPGKDKLGNYPPIPTRKGSSGKTIAALAVAALAGVSLLVGIIYVGIYRKKEQKVAANIPASSGQYHPPSPGLSGIHVDKSVEFSYEELAKSTDNFSISNKIGEGGFGTVYYAELRGKKAAIKRMNREGRTEFLAELIILTRVHHLNLVSLIGYCVERSLFLVYEFIENGNLSQHLRGKDVLTWSTRVQIAMDSARGLEYIHEHTIPFYIHRDVKSANILINENFHAKIGDFGLSKLVESGNPTLNTRFMGTFGYMPPEYGHSGVISRKVDVYAFGVVLYELISSKDAVVKEDGVDEARSLVALFDEAYSHSNQIEAISRLIDPKLHDDYPLDSVYKMAQLAKACTEKNPEMRPTMKSVVVALMVLS |
| ZmCERK1 | ONM56279.1 | MLPLLLALLTGAAPTVSASGDGCRVGCPLALAAYYFSAESNLSFIASLFGIADHNKLLPYNPGIVDPNYIVTGERVSVPFPCSCLGLPADPASTFLAGSLPYTLSGGETYDDVASEFANLTTASWLGVTNADPAGKLPAAGKINVTVNCSCGDKRVSPRYGLFLTYPLWEGETLSSVAEQYGFSSPAQLELLRRYNPGIGMDRASGKGIVFVPVKDADGSYRPLESDAGTLLHFCEICFYIRDIKHRQAKFDGKVSQDEGIKVDRSIEFSYEELSDATNNFSMDHKIGQGGFGSVYYAELRGEKAAIKKMDTKASQEFLNELKILTRVHHTNLVRLIGYCVESCLFLVYEFIENGNLSQHLHGTGYEPLSWTSRVQIALDSARGLEYIHEHTVPVYVHRDIKSANILIDRDLRAKVADFGLTKLSEIGTTSQSLPSLRVVGTFGYMPPEYARYGEVSPKVDVYAFGIVLYELLSAKEAIVRSTEFTDAQGLVYLFEETLSMPNPMEALQEMIDPRLGGDYPIDSAVKIAYLAKSCTHEEPRMRPTMRSVVVALMALSSKDHELTRGHD |
| CaLYK1 | XP_016580574.1 | MSESKPTTVLTIGIILFLFYLSNFPLPVNSQCNKGCDLALASFYVWRGLNLTFISQMFSTTIPEIVSYSNKDNIPNQDSVIAGTRINIPFRCDCLDEVKVLGHKFPYKVKLGDTYGLIASNYSDLTNAEWLMKFNRYPETGIPNAVSLDVVVNCSCGDRAVSEYYGLFITYPVRAEDNLTSVALAANVSEDVIRRYNPGVDSKLDIGNGIIYIPGRDKSGNFPPLPSTSTGLSGGAIAGISIGAIVVVLLLAGLVYVRSYRKKAQKVSLLASEDHLHQSGHGPAGSTTVKAVGSDGLADGNNPGLSDITVEKSVEFTYEELATATNDFSIANKIGEGGFGAVYYAELRGEKAAIKKMDMEATREFLAELKVLTNVHHLNLVRLIGYCVEGSLFLVYEYVENGHIGQHLRGTGRDPLPWSKRVQIALDSARGLEYIHEHTVPVYIHRDIKTANILIDKSFHAKVADFGLTKLTEVGSSSLQTRLVGTFGYMPPEYAQYGDVSPKVDVYAFGVVLYELISAKQAIVKPDGSVTESKGLVALFEEVLNQPDPDEELPKLVDPRLGDDYPLDSVRKMAQLAKACTHENPLIRPSMRSVVVALMTLSSSTEDWDVGSFYGNQGMINLMSGR |
| CaLYK11 | XP_016560162.1 | MIFQENRSFKLVIGILVLNILWVGAKSQCSDGCNALASFYAWNGSNLTFISNTFSTTIKNILSYNPQITNPDIIQFKSRVNVPFSCSCIDGKFMGHHFGVQVKTRTTYPRIARLYCSNLTTVEKLQESNRYDPNNVPANSIVNVIVNCSCGNSHVSEDYGLFITYPLRPNENLVTLANDFNLPQKLLEDYNPGANFNRGSGLVFIPGRDQNGTYPPLRTSTSSKGISGGAIAGISVAAVFVVALLAACLYHICYRGGEKEKDSFPSLEPYKNSSNEHHHGPANFENPSEMSPLNKGASPEAPRIAVDKSVEFSYDELANACDNFSTAYKIGQGGFASVYYGELRGEKAAIKKMDMQATKEFLAELKVLTHVHHLNLVRLIGYCVEGSLFLVYEYIENGNLSQHLRGLVPDNEPPLPWSARVRIALDAARGLEYIHEHTVPVYIHRDIKSANILIDKNYRAKVADFGLTKLIEAEGGSLHTRLVGTFGYMAPEYGQFGDVSPKIDVYAFGVVLYELISAKQAIVRASEIATDSKGLVALFEDVLNGVDPREGICKLVDPKLGDVYPLDSVWKVALLAKTCTHENPQLRPSMRSIVVALMTISSSSADWNIGAFYENQGLAHLMSGR |
| CaLYK13 | XP_016538522.1 | MIFLNLQSKKVTRLITLVLIYLFSNCTTCYSSCMNGCDIALASLFIWPEVYLPVISQLFNNISYSDILEWNNNITSTFIPTATRVNVAFPCDCLNNGEYLGHVFTYNVRANDTYDLIVTRRYSSLTNKESLMRDNSYPDNSFQDNNVTLKVTVNCSCGNKDVSRDYGLFITYPMRPGENLESIALATNTSSRLIEMYNPKVNFSAGSGLLYIPGKDKLGNYPPVPTRKGSSGKIIAAIAVVALAGVLLLVGIVYVGIYRKKEQKVAANIPECSGQSHPSSPAGYSGIHVDKSVDFSYRELAEATNDFCASNKIGEGGFGAVYYAELRGKKAAIKRMNREGRTEFLAELKILTRVHHLNLVSLIGYCVEKSLFLVYEFIENGNLSQHLRGEHVLPWSTRVQIALDSARGLEYIHEHTVPFYIHRDIKSANILITNNFHGKIGDFGLSKLVDSGNPTKHSRFLGTFGYMPPEYGHSPVVSRKVDVFAFGVVLYELISAKQAIVKEDGFDAPKSLVALFDEAHSHSNQIEAISRLIDPKLDDNYLFDSVYKMAQLAKACTEKSPQMRPTMKSVVVALMALSFPTEN |
| CaLYK14 | XP_016538526.1 | MRIIVSVIMYYYLFPADSSCTDGCPLALASYSYSYYGTINYDELKEVARLAAQHFSNISASEILSYPTSIEHGWDGLDTTIYNIPFSCDCLNGEYLGHVFPYTVSSGDSYGYIAEHRYSHLITAEWLMWFNNNRAVTLMVIVNCSCGNEHVSKDYGLFITYPMKPGENLKSIALNTNTSSELIQMYNPGLNFSAGTGLLYIPGRDKFGNYPPMPTSKGSSGKSITGKAVAPLAAVAGVTLLIWIMYVGFYRKKEQKVVPHISASAVQSHASGQGLFGVAIDKSVEFSYKELARSTNGFSVSNKIGEGGFGAVYYAELRGKATAIKQMNRQAKTEFLAELKILTRVHHLNLVCLIGYCVERYLFLVYEYIDNGNLSQHLRGKDTLPWSTRVQIALDSARGLEYIHEHTVPFYIHRDIKSANILIDKNFHAKIGDFGLTKLVENGNSIISTRFMGTFGYMPPEYGHSGIVSTKIDVYAFGVVLYEIISSKEAIVKEDDVDEARSLVALFGEAHDHPNSIEGISRLIDPKLKDNYPFDSVHKMAQLAKACTEKDHEMRPTMRSVVVALMALSSSTENG |
| CaLYK12 | XP_016560163.1 | MISMTAKTIILILQIITLSFLFFIFNGEAMSCGNGCDMAIASYHIWPGTNLTYISHIFNVTVPDILKYNPEITNQDSIISDTRINVPFSCDCLNGDFLGHTFVYKTVFGDTYKRVATMAFANLTTEYWLKRVNNYDPTNIPDYAMINVTVNCSCGDGHVSDDYGLFATYPLLRGENLSTVAVALGVPVELLEKFNPGLDFGSGSGIVFVPARDAQGNFPPLNTRSRGLSRGAIAGITVAAIVGATFFAVCLNFVIYRSKKIEEESFLQGSSDEHFNENFRPTNLEKITETGPLFGVVSPRPTGITVDKSVEFSYEELAKATNNFSMENKIGQGGFGAVFYGVVKGERTAIKKMDMQASKEFLAELKVLTHLHHLNLVRLIGYCVEGSLFLVYEYIENGNLGEHLRGSGWNPLSWSARVQIALDAARGLEYIHEHTVPLYIHRDIKSANILIDKDFRAKVADFGLTKLTEVGSTSFHTRLVGTFGYMPPEYAQYGDVSPKVDVYAFGVVLYELISAKEAIVKTSEVITESKGLVALFEDVLHQSGAREGLRTVVDPKLGDDYSLDSVCKVAQLAKACTHENPQLRPSMRSIVVALMTLSSSSTEDWDIGSFYENQGLVHFMSGR |
| GmLYK2 | ABQ59615.1 | MEHSFRLPVFFLLCASIAFSAESKCSRGCDLALASYYLSQGDLTYVSKLMESEVVSKPEDILSYNTDTITNKDLLPASIRVNVPFPCDCIDEEFLGHTFQYNLTTGDTYLSIATQNYSNLTTAEWLRSFNRYLPANIPDSGTLNVTINCSCGNSEVSKDYGLFITYPLRPEDSLQSIANETGVDRDLLVKYNPGVNFSQGSGLVYIPGKGLAGGVIAGISIGVVTGLLLLAFCVYVTYYRRKKVWKKDLLSEESRKNSARVKNVPLSDEASGDSAAEGGTNTIGIRVNKSAEFSYEELANATNNFSLANKIGQGGFGVVYYAELNGEKAAIKKMDIQATREFLAELKVLTHVHHLNLVRLIGYCVEGSLFLVYEYIENGNLGQHLRKSGFNPLPWSTRVQIALDSARGLQYIHEHTVPVYIHRDIKSENILIDKNFGAKVADFGLTKLIDVGSSSLPTVNMKGTFGYMPPEYAYGNVSPKIDVYAFGVVLYELISGKEALSRGGVSGAELKGLFDEVFDQQDTTEGLKKLVDPRLGDNYPIDSVCKMAQLARACTESDPQQRPNMSSVVVTLTALTSTTEDWDIASIIENPTLANLMSGK |
| GmLYK2b | XP_003555584.1 | MEALRLAYLLLPWWLVFSTAESACKEGCGVALGSYYLWRGSNLTYISSIMASSLLTTPDDIVNYNKDTVPSKDIIIADQRVNVPFPCDCIDGQFLGHTFRYDVQSQDTYETVARSWFANLTDVAWLRRFNTYPPDNIPDTGTLNVTVNCSCGNTDVANYGLFVTYPLRIGDTLGSVAANLSLDSALLQRYNPDVNFNQGTGLVYVPGKGLTGRAIAGIAVGIVAALLLLGVCIYVGYFRKKIQKDEFLPRDSTALFAQDGKDETSRSSANETSGPGGPAIITDITVNKSVEFSYEELATATDNFSLANKIGQGGFGSVYYAELRGEKAAIKKMDMQASKEFLAELNVLTRVHHLNLVRLIGYSIEGSLFLVYEYIENGNLSQHLRGSGSREPLPWATRVQIALDSARGLEYIHEHTVPVYIHRDIKSANILIDKNFRGKVADFGLTKLTEVGSSSLPTGRLVGTFGYMPPEYAQYGDVSPKVDVYAFGVVLYELISAKEAIVKTNDSVADSKGLFDGVLSQPDPTEELCKLVDPRLGDNYPIDSVRKMAQLAKACTQDNPQLRPSMRSIVVALMTLSSTTDDWDVGSFYENQNLVSFGEGEGIDVHCCSGWFVDGGIRDGQISMDVYDTIMSEEVDMDDQNQQECGVNEPHVTFNTSQVFGSRDDVLQWARSIAHESGFVAVIIRSDTNTSSRGRTSFVLIGSERSGEYRCRKKEFVRRDTGTRKCECPFKLCDKPVVGGQGWMVKLMCGIHNHELAKSLVGHPYAGRLTKAEKTLIADMTKSMVKPRNILLTLKEHNVSSCKTIKQIYNARSAYRSSIRGSDTKMQHLMKLIERDQ |
| GmLYK3 | KYP62732.1 | MTTHPTTKSKPPHVFFLLLIQLLISITRVKGSCVTGCNLALASYYLGNGTNLTYISNLFGRPTSEILKYNPSVKNPNVILSQTRINVPFSCDCLNGAFLGHTFSYAIQHGNTYKIVAEVDFSNLTTEDWVGRVNSYPPNQIPDNVNINVTVNCSCGNRHVSKDYGLFMTYPLRVGDSLQRVAAEAGVPAELLLRYNPTADFGAGNGLVFVPAKVEDQCTLHLWMTLGPALVVFWKSGGMICKGWGHSLHPYPIMYFHFHLLYTNGPSTKTQHMKNGNFPPMQLRSGISSGAIAGIAVGGAVGVLILALLLYVGLRRRRKVAEVSLLPVPGASEDQCSPLQLHHGIGCGSSLDKASESSVVASPRLTGITVDKSVEFPYEELDKATDGFSAANIIGRGGFGSVYYAELRNEVRLIGYCVEGSLFLVYEYIENGNLSQHLRGSGRDPLTWAARVQIALDAARGLEYIHEHTVPVYIHRDIKSANILIDKNFRAKFEEVLGLSDPKVDLRQLIDPTLGDNYPLDSVFKVSQLAKACTHENPQLRPSMRSIVVALMTLSSATEDWDVGSFYENQALVHLMSGR |
| GmNFR1a | ABQ59616.1 | MELKKGLLVFFLLLECVCYNVESKCVKGCDVAFASYYVSPDLSLENIARLMESSIEVIISFNEDNISNGYPLSFYRLNIPFPCDCIGGEFLGHVFEYSASAGDTYDSIAKVTYANLTTVELLRRFNGYDQNGIPANARVNVTVNCSCGNSQVSKDYGMFITYPLRPGNNLHDIANEARLDAQLLQRYNPGVNFSKESGTVFIPGRDQHGDYVPLYPRKTGLARGAAVGISIAGICSLLLLVICLYGKYFQKKEGEKTKLPTENSMAFSTQDGTVSGSAEYETSGSSGTASATGLTGIMVAKSMEFSYQELAKATNNFSLENKIGQGGFGAVYYAELRGEKTAIKKMDVQASTEFLCELKVLTHVHHFNLVQHPSNNPKHVRLIGYCVEGSLFLVYEYIDNGNLGQYLHGTGKDPLPWSGRVQIALDSARGLEYIHEHTVPVYIHRDVKSANILIDKNIRGKVADFGLTKLIEVGGSTLHTRLVGTFGYMPPEYAQYGDISPKVDVYAFGVVLYELISAKNAVLKTGESVAESKGLVALFEEALNQSNPSESIRKLVDPRLGENYPIDSVLKIAQLGRACTRDNPLLRPSMRSIVVALMTLSSPTEDCDTSYENQTLINLLSVR |
| GmNFR1b | XP_006595821.2 | MELKKWLLFFLLLEYVCCNAESKCVKGCDVALASYYVSPGYLLLENITRLMESIVLSNSDVIIYNKDKIFNENVLAFSRLNIPFPCGCIDGEFLGHVFEYSASAGDTYDSIAKVTYANLTTVELLRRFNSYDQNGIPANATVNVTVNCSCGNSQVSKDYGLFITYLLRPGNNLHDIANEARLDAQLLQSYNPGVNFSKESGDIVFIPGKDQHGDYVPLYPRKTAGLATSASVGIPIAGICVLLLVICIYVKYFQKKEGEKAKLATENSMAFSTQDVSGSAEYETSGSSGTASTSATGLTGIMVAKSMEFSYQELAKATNNFSLENKIGQGGFGIVYYAELRGEKTAIKKMDVQASTEFLCELKVLTHVHHLNLVRLIGYCVEGSLFLVYEYIDNGNLGQYLHGTGKDPFLWSSRVQIALDSARGLEYIHEHTVPVYIHRDVKSANILIDKNFRGKVADFGLTKLIEVGGSTLQTRLVGTFGYMPPEYAQYGDISPKVDVYAFGVVLYELISAKNAVLKTVESVAESKGLVALFEEALNQSNPSESIRKLVDPRLGENYPIDSVLKIAQLGRACTRDNPLLRPSMRSIVVALLTLSSPTEDCYDDTSYENQTLINLLSVR |
| CacLYK3 | XP_020222022.1 | MSTAHVFVMFELMIIIITRVEGRCFSGCSLALASYYIREGIDLTYISNLFGRPTSEIMKYNPNVTNPDQISSETRIKVPFSCDCLNGEFLGHTFSYATQHGDTYKIIAKDDFSNLTSQDWVSRVNNYPPNDIPASVNINVTVNCSCGNPHVSKDYGLFITYPLRLGDDLQGLAAESGVPAELLLRYNPNSDFSAGHGLVFLPAKDENGSFPPMQLGSSGMSSGAIAGVAVGGAAGILILALLIYVGLNRRRKVAEAPLLPLPGASGDHCSPLNHGIGIGSSLDKTSESSAVASPRLTGITVDKSVEFSYEELAKATDGFSVGNIIGQGGFGSVYYAELRNEVCHHVYVRMDMQASNEFLAELKVLTHVHHLNLVRLIGYCVEGSLFLVYEYIENGNLSQHLRGSGSEYSSFVGRDPLTWAARVQIALDAARGLEYIHEHTVPVYIHRDIKSANILIDKNFRAKVADFGLAKLTEYGSSSLQTRLVGTFGYMPPEYAQYGEVSPKIDVYAFGVVLYELISGKEAIVKTNESENDSKGLVALFEEVPGLPDSKEDLRQLVDPRLGDNYPLDSVLKVSQLAKACTHENPQLRPSMRSIVVALMTLSSSTEDWDVGSFYENQALVHLMSGR |
| CacLYK1 | XP_020213700.1 | MCVKECDVALASYYILPGYLTVENVTGWLESSVLSNSDVIKSYNKDKIIKDNLPSFDRINVPFPCDCIHEEFLGHVFEYSAAAGDTYDSIAKVTYANLTTVELLTRFNSYGHDIPQNAKINVTVKCSCGNSQVSKDYGLFITYPLRPGNNLHDIANEVHLDAQLLEKYNPGVNFSKDSGIVFIPGRDQNGDYVPLYPRKAGGIKKKISAKDAAVGVSIAGICGLLLLVVCIYVKYLQKKEGEEVKLPTESSMAFSTQDGTASGSSEYETSGSSGPTTASATGLTGIMVAKSMEFSYQELAKATNNFSLENKIGQGGFGAVYYAELRGEKTAIKKMDVQASTEFLCELKVLTHVHHLNLVRLIGYCVEGSLFLVYEYIDNGNLGQYLHGTGKDPLPWSSRVQIALDSARGLEYIHEHTVPVYIHRDVKSANILIDKNFRGKVADFGLTKLIEVGGSTLHTRLVGTFGYMPPEYAQYGDVSPKVDVYAFGVVLYELISAKNAILRTGESVAETKGLVALFEEALNQSNPGEVLSQPKLKVFWLFDELQIAQLGRACTRDNPLLRPSMRSIVVALMTLSSPTEDCEDGASYENQTLINLLSVR |
| CacLYK4 | CDH30704.1 | MENRFRLPLFFLLLATTSFSAESKCRRVCDLALASYYLPEGNLTYITDLMKSQLLSEAEDIVSYNKDIESKDQVPASTRVNVPFPCDCINGEFLGHIFQYNLAVGDTYTSIANQNYSNLTTDKWLQSFNSYSPTNIPDSGTLNVTINCSCGNSDVSKDYGLFITYPLRPEDSLQSIANKTGIKSELLLEYNPGVNFSQGSGLVYIPGKDQNDSYPPLHLKVTQGKDILKVQCKPTFSSYIVSAKAGLAGGVIAGISVGVVTGLLLLLAFCVYVIYYRRKMLWRKKLLSEESRMKSTRVKDDEATGDPSAEGGNNTTGIRVDKSAEFTYEELVNATNNFSLANKIGQGGFGEVYYAELKGEQAAIKKMDMQASGQFLAELKVLTRVHHLNLVRLIGYCVKDSLFLVYEFIENGNLGQHLRKSGFDPLPWSTRVQIALDSARGLQYIHEHTVPVYIHRDIKSENILIDKNFCAKVADFGLTKLIDVGSSSLPTDNMKGTFGYMPPEYAYGNVSPKIDVYAFGVVLYELISAKEAVIKGPELKGLVSLFDEVFEQEDPTEGLKKLVDPRLGDNYPIDSVCKLRPFYLMQMAHLARACTESDPQQRPSMSSVVVNLTALISTTEDWDIASIIENSTLVNLMSGK |
| CacLYK2 | XP_020220445.1 | MEPRFRFLLILVLLVLVLVIVPLEVESACKQGCPVALGSYYMWSGSKLTYISQIMPSALLTKPEDIVAYNKDTVPNKDSVQAFIRVNVPFPCDCVDQQFLAHTFQYDVQSQDTYDYVARTVFANLTDVAWLRRFNSYAPDNIPDTGTLNVTVNCSCGNSDVGGYGLFVTYPLRPGDTLGSVASSVGLDSGLLQRYNPGVNFNQGTGLVYIPGKDQNGSYVFLSSSSGGLPGGAIAGIAVGVVAGLLLLGVCLYVGYFRKKKIRKVELPLDSSALFAQDGKGIVISAYTPSYASLYLVPNPLYRVNSNVLKNETSRSSANETSGPGGPAAITGITVDKSVEFTYEELATATDNFSLANKIGQGGFGSVYYAELRGEVESYSKRIFRDFNDGSSINLGIDLGFEFSHTAIRVRLIGYSIEGSLFLVYEYIENGNLSQHLRGAGSREPLPWATRVQIALDSARGLEYIHEYTVPVYIHRDIKSANILIDKNFRGKVADFGLTKLTEVGSSSLPTGRLVGTFGYMPPEYAQYGDVSPKVDVYAFGVVLYELISAKEAIVKTSESEADSKGLVALFDGVLSQPDPTEDLCKLVDPRLGDNYPIDSVRKMAQLGKACTQDNPQLRPSMRSIVVALMTLSSTTDDWDVGSFYENQNLVNLMSGR |
| LjLYS1 | CAN88848.1 | MEPKLTFSLSFLLTLLSPFAESKCIKGCDLALASYYQWSGSNLTYISKIMESQILSKPQDIVTYNKGKRNFGVFSTRVNVPFPCDCINGEFLGHTFEYQLQPEETYTTVASETFSNLTVDVWMQGFNIYPPTNIPDFAVLNVTVNCSCGNSEVSKDYGLFITYPLRIEDSLQSIAEEMKLEAELLQRYNPGVNFSQGSGLVFIPGKDQNGSYVPFQQSTVGFSGGVIAGISVGVLVGLLLVAFCVYTKHLQKKKALEKKLILDDSTVNSAQVSNDSGGIMMDKSREFSYKELADATNNFSVANRIGEGGFGTVYYADLSGEKTAIKKMNMLASREFLAEVKVLANVHHLNLVRLIGYCIEGSLFLVYEYIDNGNLKQSLHDLEREPLPWSTRVQIALDSARALEYIHEHTVHVYIHRDIKSENILLDNSFHAKVADFGLSKLVQVGNSIGSSVNMMKGTFGYMPPEYARGVVSPSPKIDVYAFGVVLYELISAKEAVIRDGAQSKGLVALFDEVLGNQLDPRESLVSLVDPRLQDNYSIDSVCKMAQLAKVCTERDPTGRPSMRSVMVALMTLSSTTQSWDIASFYENPALVNRMSGRLE |
| LjLYS2 | BAI79268.1 | MDLKSRLTFFFLLSWACISFSVVESMCISGCDLALASYYIWIGSNLTYISNIMESRVLSEPEDIINYNQDHVRNPDVLQVHTRVNVPFPCDCINGEFLGHIFLHEFHEGDTYPSVAGTVFSNLTTDAWLQSTNIYGPTSIPVLAKVDVTVNCSCGDIKVSKDYGLFITYPLRAEDTLESIAEEAKLQPHLLQRYNPGVDFSRGNGLVFIPGKDENGVYVPLHIRKAGLARVVAGVSIGGTCGLLLFALCIYMRYFRKKEGEEAKFPPKESMEPSIQDDSKIHPAANGSAGFKYIMMDRSSEFSYEELANATNDFNLAKIGQGGFGEVYYAELRGEKVAIKKMKIQASREFLAELKVLTSVHHLNLVRLIGYCVERSLFLVYEYMDNGNLSQHLRESERELMTWSTRLQIALDVARGLEYIHDYTVPVYIHRDIKPDNILLNKNFNAKVADFGLTKLTDIESSAINTDHMAGTFGYMPPENALGRVSRKIDVYAFGVVLYELISAKEAVVEIKESSTELKSLEIKTDEPSVEFKSLVALFDEVIDHEGNPIEGLRKLVDPRLGENYSIDSIREMAQLAKACTDRDPKQRPPMRSVVVVLMALNSATDDRMSHAEVNSSRAGALSPTVESL |
| LjLYS6 | BAI79273.1 | MEHPRLGFPITLLLFSFILLPSTSQSKCTHGCALAQASYYLLNGSNLTYISEIMQSSLLTKPEDIVSYNQDTIASKDSVQAGQRINVPFPCDCIEGEFLGHTFQYDVQKGDRYDTIAGTNYANLTTVEWLRRFNSYPPDNIPDTGTLNVTVNCSCGDSGVGDYGLFVTYPLRPGETLGSVASNVKLDSALLQKYNPNVNFNQGSGIVYIPAKDQNGSYVLLGSSSGGLAGGAIAGIAAGVAVCLLLLAGFIYVGYFRKKRIQKEELLSQETRAIFPQDGKDENPRSTVNETPGPGGPAAMAGITVDKSVEFSYDELATATDNFSLANKIGQGGFGSVYYAELRGERAAIKKMDMQASKEFLAELKVLTRVHHLNLVRLIGYSIEGSLFLVYEFIENGNLSQHLRGSGRDPLPWATRVQIALDSARGLEYIHEHTVPVYIHRDIKSANILIDKNYRGKVADFGLTKLTEVGSSSLPTGRLVGTFGYMPPEYAQYGDVSPKVDVYAFGVVLYELISAKDAIVKTSESITDSKGLVALFEGVLSQPDPTEDLRKLVDQRLGDNYPVDSVRKMAQLAKACTQDNPQLRPSMRSIVVALMTLSSTTDDWDVGSFYENQNLVNLMSGR |
| LjLYS7 | BAI79274.1 | MTTKPNRAFFLIYFLFLLLIIIKAQGSCVSGCNLALASYTIWQGANLTYISKLFGKEPSEIMKYNPNVKNPDVIQSETQINVPFSCECLDGIFQGHTFSYTMQAGNTYKSIAKVDFSNLTTEEWVTRVNRYKPNDIPIGVKINVTINCSCGDERVSKGYGLFLTYPLRPGDDLPRLAVESGVSAEVLQGYNAGADFSAGNGLVFLPAKDENGNFPPLQKLGRSGISPGAIAGIVVGGAVVILLLAFASYVGLNRRTKVDEVSLLPVPGSYEDHNSQQLHHGCGSSMYKASESSTVVSPRLTGITVDKSVEFPYEELAKATDSFSNANIIGRGGFGSVYYAELRNEKAAIKKMDMQASNEFLAELKVLTHVHHLNLVRLIGYCVEGSLFLVYEYIENGNLSEHLRGSGRDPLSWPARVQIALDSARGLEYIHEHTVPVYIHRDIKSANILIDKNFRGKVADFGLTKLTEYGSSSLQTRLVGTFGYMPPEYAQYGEISPKVDVYAFGVVLYELVSGKEAIVRTNGPENESKALIALFEEVLGQPDPKEYLGKLVDPRLGDSYPLDSVFKVSQLAKACTHENPQLRPSMRSIVVALMTLTCAAEDWDVGSFYENQALVHLMSGR |
| LjNFR1 | CAE02590.1 | MKLKTGLLLFFILLLGHVCFHVESNCLKGCDLALASYYILPGVFILQNITTFMQSEIVSSNDAITSYNKDKILNDINIQSFQRLNIPFPCDCIGGEFLGHVFEYSASKGDTYETIANLYYANLTTVDLLKRFNSYDPKNIPVNAKVNVTVNCSCGNSQVSKDYGLFITYPIRPGDTLQDIANQSSLDAGLIQSFNPSVNFSKDSGIAFIPGRYKNGVYVPLYHRTAGLASGAAVGISIAGTFVLLLLAFCMYVRYQKKEEEKAKLPTDISMALSTQDGNASSSAEYETSGSSGPGTASATGLTSIMVAKSMEFSYQELAKATNNFSLDNKIGQGGFGAVYYAELRGKKTAIKKMDVQASTEFLCELKVLTHVHHLNLVRLIGYCVEGSLFLVYEHIDNGNLGQYLHGSGKEPLPWSSRVQIALDAARGLEYIHEHTVPVYIHRDVKSANILIDKNLRGKVADFGLTKLIEVGNSTLQTRLVGTFGYMPPEYAQYGDISPKIDVYAFGVVLFELISAKNAVLKTGELVAESKGLVALFEEALNKSDPCDALRKLVDPRLGENYPIDSVLKIAQLGRACTRDNPLLRPSMRSLVVALMTLSSLTEDCDDESSYESQTLINLLSVR |
| BnLYK1 | CDY44321.1 | MELRIRIATLLPLLLLLFSTSSLFFTVESKCSGSCNLALASYFLENGTTLSNINQNLNSPAAPFDQVNFEPILRYNPSITNKDLIQMGSRVLVPFPCECQPGDFLAHVFRYSIQQEDTYGVVATKHYANLTTEESLQRTNSFPATNIPPSATLNVSVNCFCGNESVSKDYGLFVTYPLRPEDSLDAIASSSGVPAEFIQRYNPGVDFRSGRGIVFVPGKDPNGTFPPFNSSDKAGGLGAGVIAGICIGVLVALLLISFVIYYAYRKNKKQDSHSSSIPLSAKVDQASLQSGDLVGTGVAPGLAAISVDKSVEFTLEELAKATDNFNLSFKIGQGGFGAVYYAELRGEKAAIKKMDMEASKQFLAELKVLTRVHHVNLVRLIGYCVEGSLFLIYEYVENGNLGQHLHGSGREPLPWIKRVHIALDSARGLEYIHEHTVPVYVHRDIKSANILIDQSFRAKVADFGLTKLTEVGSSATRGAMGTFGYMAPEIVYGEVSAKVDVYAFGVVLYELISAKAAVVKMNQASGEFRGLVGVFEEVFKEADKEEALRKIIDPRLGDNYPFDSVYKMAELGKACTQENAQLRPSMRYIVVALSTLISSTGNWDVGNFQNDDIVSLMSGR |
| BrLYK1 | XP_009145469.1 | MELRIQIATLLLLLFWPSSLFFTVESKCSGSCNLALASYFLDNGTTLSNINLNLNSPEAPYDQINFEPILRYNPSITNKDLIQMGTRLLVPFPCECQPGDFLAHVFRYSVQPEDTYDIVATEHYANLTTEESLRRTNSFPATNIPPSATLNVSVNCFCGNESVSKDYGLFVTYPLRPEDSLDAIASSSGVPAETLQRYNPGVDFRSGSGIVFVPGKDPNGTFPPFKSSDKGGLGAGVIAGICIGVLVALLLISFVIYYVYRKNKKQESHSSSIPLSAKVDQASLQSGDLVGTGVAPGLAAISVDKSVEFTLEELAKATDNFNLSFKIGQGGFGAVYYAELRGEKAAIKKMDMEASKQFLAELKVLTRVHHVNLVRLIGYCVEGSLFLIYEYVENGNLGQHLHGSGREPLPWTKRVHIALDSARGLEYIHEHTVPVYVHRDIKSANILIDQNFRAKVADFGLTKLTEVGSSATRGAMGTFGYMAPEIVYGEVSAKVDVYAFGVVLYELISAKAAVVKMNQASGEFRGLVGVFEEVFKEADKEEALRKIIDPRLGDNYPFDSVYKMAELGKACTQENAQLRPSMRYIVVALSTLFSSTGNWDVGNFQNDDIVSLMSGR |
| LaLYK1 | OIW17089.1 | MFLLWLLLLSSAESKCTQGCSLALASYYMYSGSTLTSISQVMSSQLLQIPEDIVTYNKDTIPNKDSVQAFIRVNVPFPCDCIDGEFLGHMFQYDVKTGDTYQLVAETEYANLTNIDWLMKFNSYPANNIPDTGTLNVTVNCSCGEKNVSNYGLFITYPLRPGDTLDSVSKSVDLDSGLLQRYNPGVNFNQGSGLVYIPGKDQNGSYVFLNSSSEGLAGGVIAGIVIGVLAGILLLVAGIYVGYFRKKKIQKEELLEQDSKSLFVQNDETARTAATTGISVDKSVEFSYEELASATDNFSMANKIGQGGFGVVYYAELRGEKAAIKKMDMQASKEFLAELKVLTHVHHLNLVRLIGYSIEGSLFLVYEFIENGNLSQHLRGSGRDPLPWPARVQIALDSARGLEYIHEHTVPVYIHRDIKSANILIDKNFRGKLAQLAKACTQDNPQLRPSMRSIVVALMTLSSTTDDWDVGSFYENQNLVNLMSGR |
| LaLYK2 | XP_019434083.1 | MKLKLVFLLLLKYVCFIVESKCIKGCDLALASYYVPVWPIVSLGNITSFMHSNVLTNPNVVTSYNKDKVFNGDVMLALYRTNVPFPCDCIGGEFLGHVFEYSAVEGDTYGLIAMKRYSNLTTVEILKRFNSYDPNHIPVNAKVNVTVKCSCGNSQISKDYGLFITYPLRPGNNLQELSKETKIDAKLLQSYNPGVNFSQENGIVFIPGKDQNGVYVPLYPRTGGVAKGVAVGISIAATCGLVLLVICIYDRYFKKKEGEKAKLSIENSIGFSTQDAYGSGEYETSGSSVHASALTGIMVAKSLEFSYQELAKATNNFSLDNKIGQGGFGAVYYAELRGEKTAIKKMDVQASSEFLAELKVLTHVHHLNLVRLIGYCVEGSLFLVYEYIDNGNLGQYLHGKGKDPLPWSTRLQIALDSARGLEYIHEHTVPLYIHRDVKSANILIDKNLRGKVADFGLTKLIEVGTSSLHTRLVGTFGYMPPEYAQYGDISPKIDVYAFGVVLYELISAKNAVLKTGETVAESKGLVNLFEEALNQINPLEPLTTLVDPRLGDNYPIQSLLKIAELGRACTRDNPLLRPNMKSIVVALMTLSSSNEDNTTSSYDNQTLINLLLDEGFRGITF |
| GsNFR1a | KHM99103.1 | MELKKWLLFFLLLEYVCCNAESKCVKGCDVALASYYVSPGYLLFENITRLMESIVLSNSDVIIYNKDKIFNENVLAFSRLNIPFPCGCIDGEFLGHVFEYSASAGDTYDSIAKVTYANLTTVELLRRFNSYDQNGIPANATVNVTVNCSCGNSQVSKDYGLFITYPLRPGNNLHDIANEARLDAQLLQSYNPSVNFSKESGDIVFIPGRDQHGDYVPLYPRKTGLATSASVGIPIAGICVLLLVICIYVKYFQKKEGEKAKLATENSMAFSTQDVSGSAEYETSGSSGTASTSATGLTGIMVAKSMEFSYQELAKATNNFSLENKIGQGEFGIVYYAELRGEKTAIKKMDVQASTEFLCELKVLTHVHHLNLVRLIGYCVEGSLFLVYEYIDNGNLGQYLHGTGKDPFLWSSRVQIALDSARGLEYIHEHTVPVYIHRDVKSANILIDKNFRGKVADFGLTKLIEVGGSTLQTRLVGTFGYMPPEYVQYGDISPKVDVYSFGVVLYELISAKNAVLKTGESVAESKGLVALFEEALNQSNPSESIRKLVDPRLGENYPIDSVLKIAQLGRACTRDNPLLRPSMRSIVVALMTLSSPTEDCYDDTSYENQTLINLLSVR |
| GsNFR1b | KHN38471.1 | MELKKGLLVFFLLLECVCYNVESKCVKGCDVAFASYYVSPDLSLENIARLMESSIEVIISFNEDNISNGYPLSFYRLNIPFPCDCIGGEFLGHVFEYSASAGDTYDSIAKVTYANLTTVELLRRFNGYDQNGIPANARVNVTVNCSCGNSQVSKDYGMFITYPLRPGNNLHDIANEARLDAQLLQRYNPGVNFSKESGTVFIPGRGLARGAAVGISIAGICSLLLLVICLYGKYFQKKEGEKTKLPTENSMAFSTQDVSGSAEYETSGSSGTASATGLTGIMVAKSMEFSYQELAKATNNFSLENKIGQGGFGAVYYAELRGEKTAIKKMDVQASTEFLCELKVLTHVHHFNLVRLIGYCVEGSLFLVYEYIDNGNLGQYLHGTGKDPLPWSGRVQIALDSARGLEYIHEHTVPVYIHRDVKSANILIDKNIRGKVADFGLTKLIEVGGSTLHTRLVGTFGYMPPEYAQYGDISPKVDVYAFGVVLYELISAKNAVLKTGESVAESKGLVALFEEALNQSNPSESIRKLVDPRLGENYPIDSVLKIAQLGRACTRDNPLLRPSMRSIVVALMTLSSPTEDCDTSYENQTLINLLSVR |
| GsLYK1 | KHN46333.1 | MASSLLTTPDDIVNYNKDTVPSKDIIIADQRVNVPFPCDCIDGQFLGHTFRYDVQSQDTYETVARSWFANLTDVAWLRRFNTYPPDNIPDTGTLNVTVNCSCGNTDVANYGLFVTYPLRIGDTLGSVAANLSLDSALLQRYNPDVNFNQGTGLVYVPGKDQNGSFVRLPSSSGGLTGRAIAGIAVGIVAALLLLGVCIYLGYFRKKIQKDEFLPRDSTALFAQDGKDETSRSSANETSGPGGPAIITDITVNKSVEFSYEELATATDNFSLANKIGQGGFGSVYYAELRGEKAAIKKMDMQASKEFLAELNVLTRVHHLNLVRLIGYSIEGSLFLVYEYIENGNLSQHLRGSGSREPLPWATRVQIALDSARGLEYIHEHTVPVYIHRDIKSANILIDKNFRGKVADFGLTKLTEVGSSSLPTGRLVGTFGYMPPEYAQYGDVSPKVDVYAFGVVLYELISAKEAIVKTNDSVADSKGLVALFDGVLSQPDPTEELCKLVDPRLGDNYPIDSVRKMAQLAKACTQDNPQLRPSMRSIVVALMTLSSTTDDWDVGSFYENQNLVNLMSGR |
| GsLYK2 | KHN32998.1 | MTTHPTTKSKPPHVFFLLLIQLLISITRVKGSCVTGCNLALASYYLGNGTNLTYISNLFGRPTSEILKYNPSVKNPNVILSQTRINVPFSCDCLNGAFLGHTFSYAIQHGNTYKIVAEVDFSNLTTEDWVGRVNSSPPNQIPDNVNINVTVNCSCGNRHVSKDYGLFMTYPLRRVAAEAGVPAELLLRYNPTADFGAGNGLVFVPAKDENGNFPPMQLRSGISSGAIAGIAVGGAVGVLILALLLYVGLRRRRKVAEVSLLPVPGASEDQCSPLQLHHGIGCGSSLDKASESSVVASPRLTGITVDKSVEFPYEELDKATDGFSAANIIGRGGFGSVYYAELRNEKAAIKKMDMQASNEFLAELNVLTHVHHLNLVRLIGYCVEGSLFLVYEYIENGNLSQHLRGSGRDPLTWAARVQIALDAARGLEYIHEHTVPVYIHRDIKSANILIDKNFRAKVADFGLTKLTEYGSSSLHTRLVGTFGYMPPEYAQYGDVSSKIDVYAFGVVLYELISGKEAIVRTNEPENESKGLVALFEEVLGLSDPKVDLRQLIDPTLGDNYPLDSVFKVSQLAKACTHENPQLRPSMRSIVVALMTLSSATEDWDVGSFYENQALVHLMSGR |
